# Investigating the Structure and Dynamics of the *PIK3CA* Wild-Type and H1047R Oncogenic Mutant

**DOI:** 10.1371/journal.pcbi.1003895

**Published:** 2014-10-23

**Authors:** Paraskevi Gkeka, Thomas Evangelidis, Maria Pavlaki, Vasiliki Lazani, Savvas Christoforidis, Bogos Agianian, Zoe Cournia

**Affiliations:** 1 Biomedical Research Foundation, Academy of Athens, Athens, Greece; 2 Department of Molecular Biology and Genetics, Democritus University of Thrace, Alexandroupolis, Greece; 3 Department of Biomedical Research, Institute of Molecular Biology and Biotechnology-Foundation for Research and Technology (IMBB-BR/FORTH), Ioannina, Greece; 4 Department of Medicine, University of Ioannina, Ioannina, Greece; Max Planck Institute for Biophysical Chemistry, Germany

## Abstract

The *PIK3CA* gene is one of the most frequently mutated oncogenes in human cancers. It encodes p110*α*, the catalytic subunit of phosphatidylinositol 3-kinase alpha (PI3Kα), which activates signaling cascades leading to cell proliferation, survival, and cell growth. The most frequent mutation in *PIK3CA* is H1047R, which results in enzymatic overactivation. Understanding how the H1047R mutation causes the enhanced activity of the protein in atomic detail is central to developing mutant-specific therapeutics for cancer. To this end, Surface Plasmon Resonance (SPR) experiments and Molecular Dynamics (MD) simulations were carried out for both wild-type (WT) and H1047R mutant proteins. An expanded positive charge distribution on the membrane binding regions of the mutant with respect to the WT protein is observed through MD simulations, which justifies the increased ability of the mutated protein variant to bind to membranes rich in anionic lipids in our SPR experiments. Our results further support an auto-inhibitory role of the C-terminal tail in the WT protein, which is abolished in the mutant protein due to loss of crucial intermolecular interactions. Moreover, Functional Mode Analysis reveals that the H1047R mutation alters the twisting motion of the N-lobe of the kinase domain with respect to the C-lobe and shifts the position of the conserved P-loop residues in the vicinity of the active site. These findings demonstrate the dynamical and structural differences of the two proteins in atomic detail and propose a mechanism of overactivation for the mutant protein. The results may be further utilized for the design of mutant-specific PI3Kα inhibitors that exploit the altered mutant conformation.

## Author Summary

The PI3Kα protein is involved in cellular processes such as cell growth, division, and formation of new blood vessels (angiogenesis) that aid cancer cell survival. In certain types of cancer cells, PI3Kα is found to be altered compared to healthy cells. These PI3Kα alterations, called mutations, are found in 27% of breast cancer patients, 24% of endometrial cancer patients, and 15% of colon cancer patients. PI3Kα mutations cause the protein to become overactivated and may contribute to tumor growth. The most common PI3Kα amino acid mutation is a histidine changed to an arginine: H1047R. Understanding how the H1047R mutation overactivates PI3Kα is central to developing therapeutics for cancer patients who bear PI3Kα mutations. To this end, we performed simulations and experiments of the mutated and physiological proteins to explain why the mutant protein becomes overactivated. Our results indicate structural and dynamical differences between the mutant and physiological proteins that may affect the PI3Kα function. Based on these differences, we propose a mechanism that highlights the series of events that lead to the mutant H1047R PI3Kα overactivation. This study provides insights into developing mutant-specific PI3Kα inhibitors that exploit the altered conformation of the mutant with respect to the physiological protein.

## Introduction

The PI3Kα protein is involved in cellular processes vital for cancer progression, such as cell growth, proliferation, motility, survival, and metabolism [1]. As a result, deregulation of PI3Kα signaling is one of the most frequent events leading to cancer [2]. PI3Kα uses ATP to phosphorylate the phosphatidylinositol PIP2 to PIP3, a reaction that requires prior attachment of the enzyme to the cell membrane. Increased PI3Kα signaling may occur by several mechanisms, including somatic mutations and amplification of genes encoding key components of the PI3Kα pathway [1]. PI3Kα comprises a catalytic subunit, p110α, and a regulatory subunit, p85α. The p110α subunit consists of five domains: the adaptor-binding domain (ABD), the RAS-binding domain (RBD), and the C2, helical, and kinase domains. Somatic mutations within the gene encoding p110α (*PIK3CA*) are frequently observed in a variety of human tumors, including breast, colon, endometrial cancers, and glioblastomas [3]. These mutations are scattered over the length of p110α but two hotspots account for nearly 80% of them: an H1047R substitution close to the C-terminus and a cluster of three charge-reversal mutations (E542K, E545K, Q546K) in the helical domain of p110α [4]. Both types of mutations can induce oncogenic transformation in cell cultures [5], while H1047R is also able to induce tumorigenesis in transgenic mice [6], [7]. According to structural and functional studies, these two hot spot mutations act synergistically, but independently [8]–[10].

The structure of the human [11] and mouse [8] catalytic subunit p110α has been solved by X-ray crystallography, as well as the structure of the human H1047R mutant [12]. Recent experimental data demonstrate that the H1047R mutation overactivates the enzyme by inducing dynamic changes in the kinase domain, which increase basal ATPase activity as well as expose the membrane binding regions, thereby augmenting basal membrane binding [8], [12], [13]. It has also been shown that the C-terminal region, where H1047R resides, is essential for catalysis. The C-terminus enhances membrane binding, while it inhibits the basal activity of the enzyme in the absence of the membrane [14]–[16]. This recent experimental work has provided mechanistic insights into the mutational activation; however, an atomic-level description of the factors that contribute to the enzyme overactivity is still missing.

In the present study, we have modeled the full-length catalytic p110α subunit in the WT and H1047R mutant forms in order to gain insights into the overactivation mechanism of the commonly-expressed H1047R mutant through Molecular Dynamics (MD) simulations and Functional Mode Analysis (FMA) and have used SPR experiments to validate our results. The simulations are in excellent agreement with experimental data and allow us to provide atomic-detail insights into the mechanism of overactivation of the *PIK3CA* H1047R mutant by monitoring structural and dynamical elements of the WT and mutant proteins.

## Results

### Convergence of WT and H1047R systems

Five independent simulations have been performed for the full length WT and H1047R mutant p110α (the catalytic subunit of PI3Kα), respectively (Figure S1 and Table S1). Cα Root Mean Square Deviation (RMSD) indicates that a plateau is reached in ∼100–120 ns for the WT and ∼100–135 ns for the mutant p110α (Figure S2). For the different systems, we performed Principal Component Analysis (PCA) using the last 50 ns of sampling for each independent simulation for the WT and H1047R mutant proteins, respectively. The 50 ns used for the PCA were chosen based on the RMSD of the Cα of the non-flexible loops (res. number 1–7, 231–240, 291–330, 410–417, 505–530, 863–872, 941–952, 1047–1068). The overlap of the 2D projections of the trajectories on the first two eigenvectors indicates that the five independent simulations for the WT and the H1047R mutant proteins span the same or similar conformational phase space (Figure S3). For the analysis of the trajectories we used the last 50 ns of each trajectory, i.e. WT1 (100–150 ns), WT2 (100–150 ns), WT3 (100–150 ns), WT4 (120–170 ns), WT5 (100–150 ns) for the WT and Mut1 (125–175 ns), Mut2 (100–150 ns), Mut3 (130–180 ns), Mut4 (135–185 ns), Mut5 (100–150 ns) for the mutant (Table S1).

### Cluster analysis and conformation of a second binding pocket in PI3Kα

**Figure 1:**
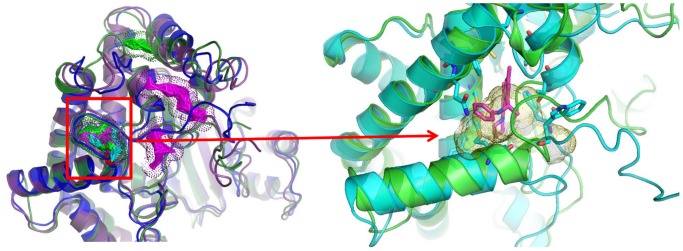
Conformation of a second binding pocket in PI3Kα. (A) The first three cluster representatives from the WT trajectory in blue, green, and magenta, respectively. Dots represent predicted binding sites identified by the Q-SiteFinder server. The color of the dots corresponds to the respective cluster representative. (B) The first cluster representative from the trajectory is colored in green and aligned with the 4A55 crystal structure in cyan. His1047 is shown in cyan stick representation. The crystallized ligand of 4A55, PIK-108, is shown in magenta [8]. The predicted binding site by QSiteFinder appears in yellow dots and highly overlaps with the position of PIK-108 in the experimental structure.

It has been proposed that a second binding site, distinct from the active site, exists in PI3Kα as demonstrated in a recent WT crystal structure [8]. Representative conformations from the first three cluster representatives of the WT protein (taken from simulation WT1, see Table S2) were submitted to Q-SiteFinder server [17] for ligand binding site prediction in the kinase domain. As seen in Figure 1, the existence of a new binding pocket, distinct from the active site and close to H1047 in the WT protein, is confirmed.

### An altered hydrogen bonding network leads to changes in polar contacts within the mutation region

**Figure 2:**
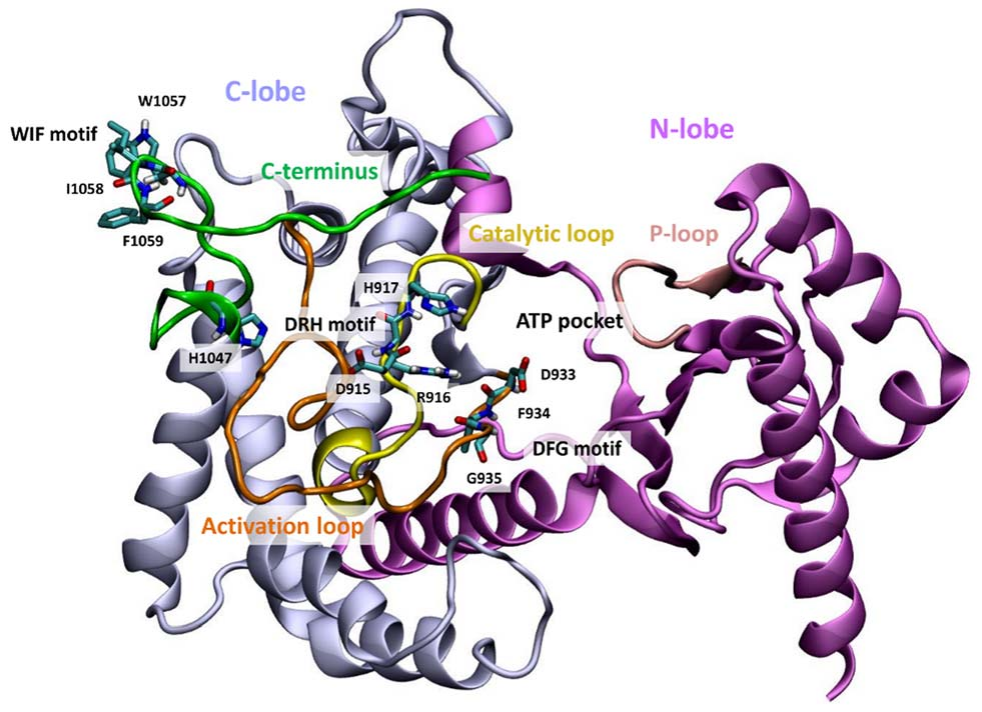
Functionally important structural elements of the PI3Kα kinase domain. Grey: C-lobe, mauve: N-lobe, yellow: catalytic loop, orange: activation loop, green: C-terminal region, pink: P-loop. In stick representation are shown the His-1047 residue, which is frequently mutated in cancer, the WIF motif residues (res. 1057–1059), the DRH motif residues (res. 915–917), and the DFG motif residues (res. 933–935).

**Figure 3:**
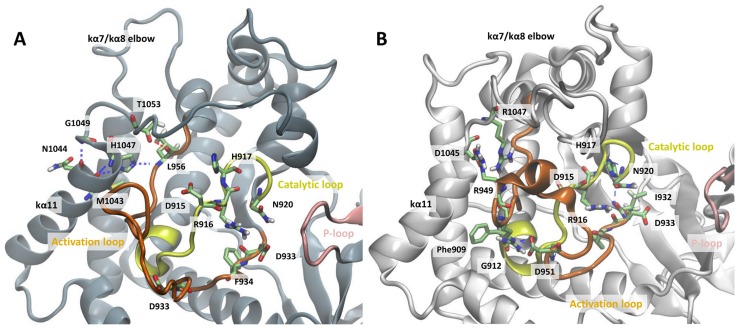
Polar contacts close to the mutation site, in (A) the WT and (B) H1047R p110α. The activation loop is colored orange, the catalytic loop yellow, and the P-loop in pink. Residues that form polar contacts are shown in licorice representation.

To determine key features of the mutant protein that may result to overactivity with respect to the WT, the structure and dynamics of interaction network in both proteins were monitored. Functionally important structural elements of the PI3Kα kinase domain are highlighted in Figure 2. It has been suggested that intermolecular interactions between helix kα11 (res. numbers 1031–1047), which precedes the C-terminal tail, and the catalytic loop (res. numbers 909–920) shield the conserved catalytic DRH motif from performing futile ATP hydrolysis by His-917 [15], [16]. Indeed, our calculations show that an interaction network, tightly coupled to His-1047, accurately controls the DRH motif and retracts it from the vicinity of the active site: the hydrogen bond between the WT His-1047 backbone nitrogen and Met-1043 backbone oxygen as well as the hydrogen bond between the WT His-1047 π-imidazole ring nitrogen (see Figure S4 for definition) and Met-1043 backbone oxygen stabilize the last α-helix of the protein, kα11 (see Figure 3 A, hydrogen bonds have frequencies 46±7% and 64±5%, respectively). Statistical errors have been calculated as standard deviations from five independent simulations. The autocorrelation functions of the hydrogen bonds time series have been calculated and found to converge within the simulation time. kα11 is further stabilized by a frequent hydrogen bond between the WT Gly-1049 nitrogen and Asn-1044 backbone oxygen (72±8%). As a result, a hydrogen bond between the His-1047 *τ*-imidazole ring nitrogen and the amide hydrogen of activation loop residue Leu-956 occurs at high frequency (92±1%) in four out of five simulations of the WT p110α, in agreement with experimental data (Table S3) [12]. In Simulation WT1, this hydrogen bond is infrequent (1%), however, Leu-956 backbone oxygen is frequently hydrogen bonded with Thr-1053 backbone oxygen (90%), leading to a similar conformation of the WT protein kα11 helix in all five simulations (Figure S5). As His-1047 is kept tightly controlled in the WT, Arg-949 forms frequent hydrogen bonds with Asp-915 of the conserved DRH motif in four out of five of WT simulations (78±6%, Table S3). In the remaining simulation (Simulation WT3), Arg949 side chain nitrogen atoms form hydrogen bonds with Asp-939 backbone oxygen with a frequency of 78%. In all five simulations, we consistently observed a hydrogen bond formed between Arg-916 of the DRH motif and Asp-933 of the DFG motif in agreement with Miller et al. [15].

In stark contrast, this extensive hydrogen bond network observed in the vicinity of the WT His-1047 is not present in the mutant structure. Arg-1047 breaks the hydrogen bond with Leu-956. Consequently, the His-to-Arg substitution at position 1047 destabilizes the kα11 α-helix by disruption of the hydrogen bonds between Arg-1047 and Met-1043 as well as Gly-1049 and Asn-1044 (Figure 3 B, Table S3). The disruption of the α-helix allows Asp-1045 to hydrogen bond with Arg-949 in three out of five mutant simulations. In the other two simulations, the final residues of kα11, Asp-1045, Ala-1046, and Arg-1047, are in an extended conformation and do not form any hydrogen bonds with the activation loop. In all five simulations of the H1047R mutant the activation loop residue Asp-951 forms frequent hydrogen bonds with either residues Gly-912, Phe-909 or Asp-939 from the activation loop. In turn, the hydrogen bond between Arg-949 and Asp-915 is abolished in the mutant H1047R p110α. This destabilization of Asp-915 allows the side-chain of His-917 of the DRH motif (which in the active PI3Kα conformation participates in the ATP hydrolysis) to point towards the active site (Figure S6). This conformation of the His-917 was also observed in Ref. 17, where His-745 of PI3Kγ in its active form points towards the active site as compared to the catalytic His-807 of Vsp34 (the primordial PI3Kα) that points away from the ATP site (see Figures 2 C and 3 of Ref. [15]). Similarly to the WT simulations, the hydrogen bond between Arg-916 of the DRH motif and Asp-933 of the DFG motif is also evident, though to a lesser extent (WT frequency, 79±6%; mutant, 50±14%).

To further probe intermolecular interactions that govern the observed differences between WT and mutant, we have calculated the average distances of the side chains of the WIF motif residues (res. numbers 1057, 1058, and 1059). This motif is conserved in class I and class II PI3Ks as a triplet of hydrophobic residues and has been proposed to be crucial for lipid binding in the C-terminal region [8]. Stacking of the hydrophobic side chains of the WIF motif is evident in our simulations as has been suggested by experiments and may play a role in lipid binding (Figures S7, S8 and Table S4) [8], [18]. In particular, we observe stacking between Ile-1058 and Phe-1059 in the largest part of the trajectory of both the mutant and WT simulations, while Trp-1057 and Ile-1058 are occasionally found within stacking distance in both proteins.

We also monitored the effect of the H1047R mutation on the solvent accessibility of kα11 and kα12 (C-terminal tail, residues 1032–1068). Our results demonstrate that residues 1032–1047 (kα11) are significantly more solvent exposed in the H0147R mutant, while residues 1048–1069 (kα12) are more solvent exposed in the WT (Table S5). In particular, residues Met-1043, Trp-1057 as well as the mutated residue 1047 have higher solvent accessible area in the mutant, whereas Met-1055 and Asp-1056 are more solvent accessible in the WT protein (Figure S9).

### The polar contacts within the active site are altered in the mutant form

**Figure 4:**
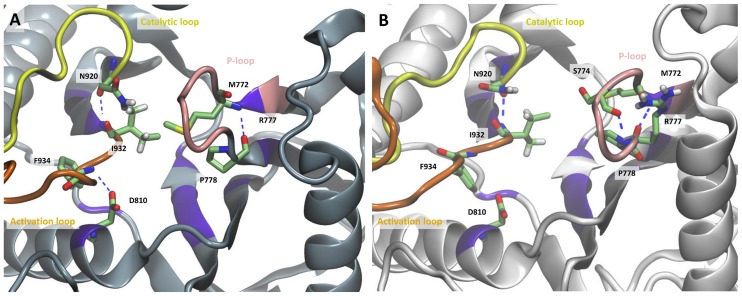
Polar contacts close to the activation site, in (A) the WT and (B) H1047R p110α. The activation loop is colored orange, the catalytic loop yellow, and the P-loop in pink. Residues that form polar contacts are shown in licorice representation.

The examination of the polar contact network of the WT and the mutant proteins (i.e. salt bridges and hydrogen bonds) indicates differences between the two protein forms within the active site (Figures 4, S10, and S11). The hydrogen bond between Asp-810 of the affinity pocket and Phe-934 of the activation loop (DFG motif) occurs at a frequency of 92±1% of the simulation time in the WT, while it has zero or low occurrence in three out of five simulations of the mutant. In the other two simulations of the mutant the Asp-810-Phe-934 hydrogen bond has an average frequency of 85±3%, this, however, does not affect the conformation of the activation loop which is similar in all five simulations of the mutant (Figure S11). Met-772 is hydrogen bonded through its backbone nitrogen to the backbone oxygen of Pro-778 in 75±12% of the simulation time in the WT and in 89±1% of the mutant trajectories. Loop residues 771–780 interact with the phosphates of the ATP and are known to comprise the P-loop (see Table 1) [18]. Despite this common interaction within the P-loop residues, two hydrogen bonds between the backbone oxygen and nitrogen of both Arg-770 and Trp-780 occur with higher frequency in the mutant protein than in the WT, 73±9% and 87±1% for the mutant and 37±8% and 39±8% in the WT, leading to a more compact conformation of the P-loop in the case of H1047R mutant (Figure 4 B). This closed conformation is further enforced through a hydrogen bond between the backbone oxygen of Ser-774 and the backbone nitrogen of Arg-777 in the mutant simulations (45±7%). The same hydrogen bond is either infrequent or completely absent in the WT simulations, allowing for a more wide conformation of the P-loop. Estimation of the solvent exposed surface area in the active site of the WT and H1047R p110α subunit showed that both proteins have similar solvent accessibility (Figure S12, Table S5). This finding is in agreement with previous observations [19].

**Table 1:** Residue numbers of functionally important structural elements of the PI3Kα kinase domain.

Functionally important elements	Residues
Mem. Bind. loop 1	721–727
P-loop	771–777
Mem. Bind. loop 2	863–873
Catalytic loop	909–920
DRH motif	915–917
Activation loop	933–958
DFG motif	933–935
C-terminus	1032–1068
WIF motif	1057–1059
Affinity pocket (ATP pocket)	810, 836, 848, 932
Adenine pocket (ATP pocket)	800, 836, 922, 930
Gatekeeper (ATP pocket)	848
Specificity pocket (ATP pocket)	772, 780
Hinge (ATP pocket)	849, 851

### H1047R accumulates positive charge in regions that contact the cell membrane

**Figure 5:**
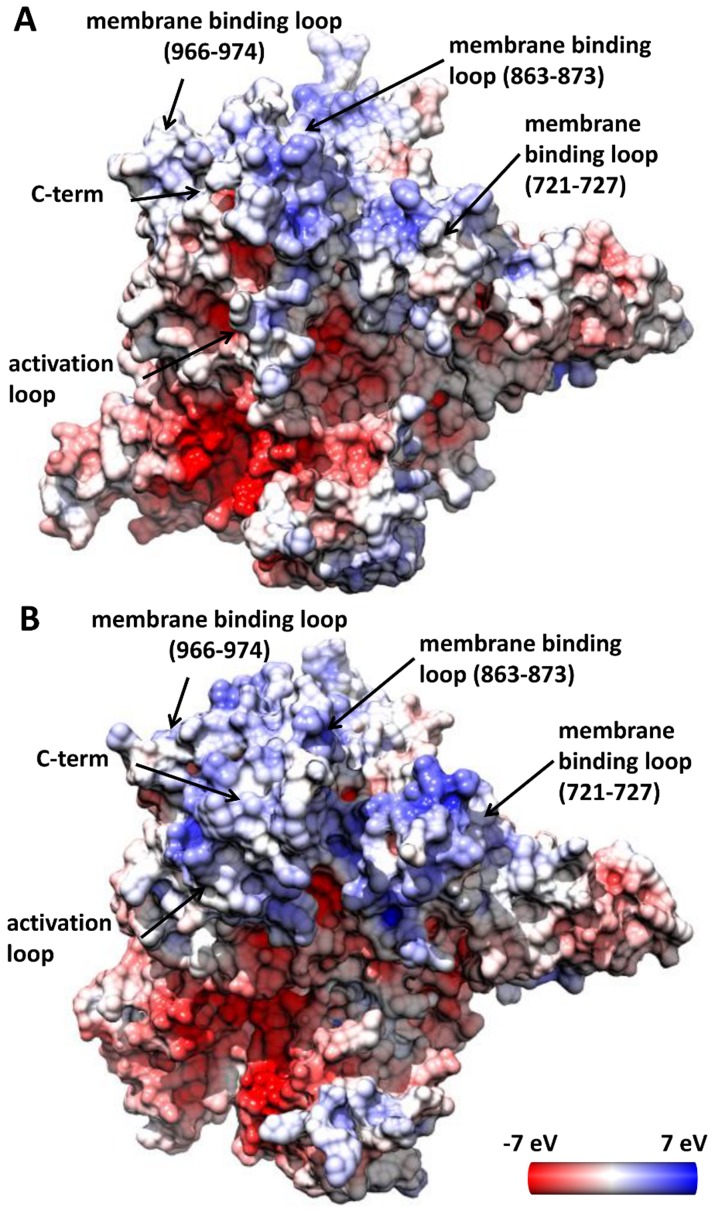
The electrostatic potential on the surface of the average conformation of the WT and H1047R p110α. The images depict membrane interaction areas of the WT (A) and H1047R mutant (B) first cluster representatives. The surface has been colored with a color scale from −7 eV to 7 eV, with red representing negative charge, white neutral and blue positive. The charge value corresponds to the solvent accessible surface of the protein, namely 1.4 Å far from the surface. These structures were derived from a cluster analysis including all the Cα carbon with a cutoff of 1.7 Å.

PI3Kα attaches to the cell membrane in order to retrieve the PIP2 substrate and transform it to PIP3. It has been reported that the H1047R mutation augments the interaction between PI3Kα and the membrane; the enzymatic activity of PI3Kα H1047R is increased compared to the WT protein upon interaction with phosphatidylserine (PS) and cancer liposomes [12]. Further lipid-PI3Kα association studies [8], using the WT and H1047R mutant proteins and neutral and anionic PS liposomes, showed that lipid binding of the mutant protein is many fold higher than that of the WT enzyme. The inner leaflet of the cell membrane has a net negative charge resulting from the predominance of phosphatidylserine and phosphatidylinositol on the cytosolic face of the plasma membrane. Thus, to rationalize the fact that H1047R PI3Kα binds to cell membranes with higher affinity, we performed electrostatic potential calculations on the proposed membrane binding domains, which involve residues 863–873, 721–727, the end of the C-terminal tail, and residues along the activation loop [11], [12]. The calculated electrostatic potential on the surface of the two proteins reveals major differences in the positive charge distribution of membrane binding regions (Figures 5 and S13). In the H1047R p110α, the C-terminus protrudes to the plane of the membrane as a hydrophobic tail, while it is surrounded by enhanced positive charge accumulated along the activation loop, the kα7/kα8 (966–974), and kα6/kα5 (863–873) elbows (Figures 5 B and S13 B,D). These regions of the kinase C-lobe, which are pronouncedly less positively charged in the WT (Figure 5 A and S13 A, B), have been found to interact with neutral and anionic membranes only in the case of H1047R mutant [13]. Interestingly, the mutant also exhibits higher positive charge than the WT along the other membrane binding site (residues 721–727 on the N-lobe) which was protected in both WT and H1047R HDX-MS experiments by PIP2 phospholipid vesicles [13]. Positive charge is also detected on loops 343–351 and 410–418 of the mutant C2 domain, which have also been proposed to contact the cell membrane [11].

### H1047R displays higher membrane binding compared to WT

**Figure 6:**
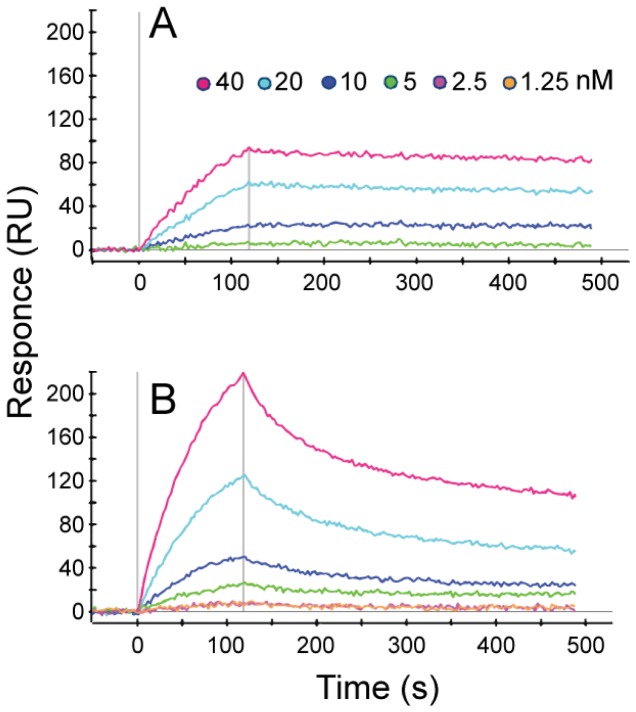
PI3Kα binding to PIP2-liposomes. Typical SPR sensograms depicting concentration dependent binding of WT p110α/p85α (A) and H1047R p110α(H1047R)/p85α (B) PI3Kα to liposomes. Signal from WT at 1.25 and 2 nM (A) was nil and is not shown for clarity. Vertical bars indicate beginning and end of injection. Data shown are referenced and corrected for bulk effect.

In order to validate our MD results and compare lipid binding of WT and mutant PI3Kα, we employed Surface Plasmon Resonance (SPR) to monitor direct binding to cancer liposomes. Liposomes were prepared from total lipid extractions from HCT116 human colorectal cancer cells and were enriched with 2% PIP2. As cell membranes contain mostly negatively charged lipids, we expect the liposomal surface to be predominantly negatively charged. In titration experiments, a concentration dependent binding of both WT (Figure 6 A) and mutated PI3Kα (Figure 6 B) to liposome surfaces was observed. H1047R bound liposomal membranes at repeatedly higher levels than equimolar WT protein (typically ∼2–2.5 fold at 40 nM at the peak of association which corresponds to end of injection; Figure 6 B). This increase in lipid binding characterizes the behavior of the inactive forms of the enzyme as no activating RTK phosphopeptide was included in the experiments. No significant binding was observed to control surfaces for both proteins, demonstrating PI3K specificity for lipids. In the absence of PIP2, we could not detect substantial PI3Kα-liposome interaction, indicating that our results do not depend on endogenous PIP2 in cancer liposomes. Comparisons were performed using data obtained from the same injection and liposomal surface, in a “one shot” grid approach (see Materials and Methods section).

### The dynamics of functionally relevant elements are significantly different in the mutant form

Root mean square fluctuation (RMSF) analysis of the MD trajectory reveals that three regions important for enzyme function exhibit different mobility in the WT and the mutant (Table S6). The activation loop (933–958) has an average RMSF of 1.26±0.12 Å in the WT and 1.66±0.16 Å in the mutant. Moreover, the catalytic loop is more flexible in the mutant with an RMSF of 1.04±0.05 Å compared to an RMSF of 0.81±0.04 Å in the WT, as well as the P-loop. The C-terminus (residues 1048–1068) sporadically forms helical turns (see Videos S1 and S2) in both the mutant and the WT proteins, but its overall flexibility remains high. The RMSF is 3.40±0.90 Å in the WT and 3.42±0.36 Å in the mutant; however their conformation is entirely different. Although the initial configurations of the C-termini of mutant and WT proteins were modeled to occupy the same area in space (Figure S14 shows the kα7/kα8 and kα6/kα5 elbows (blue) to be aligned very well in their initial conformation), during the course of the simulation the WT C-terminus always shields the ATP binding pocket, whereas the C-terminus of the mutant is pulled above the ATP site (see Videos S1 and S2).

Kinases are known to exhibit two characteristic large-scale motions in the absence of ATP: a bending motion centered at the hinge region, between the N- and the C-lobe, and a twisting motion of the N- lobe with respect to the C-lobe [20]. Although these motions can be described by Principal Component Analysis (PCA), they are not captured entirely by a single principal mode. Thereby, Functional Mode Analysis (FMA) was implemented in order to identify collective motions related to the hinge bending motion and the C- and N- lobe twisting motions. The functional quantity that yielded the highest correlation to the hinge bending motion was the distance between the C*α* carbons of Leu-781 and Met922 of the active site (*d_LM_*). Residues Leu-781 and Met-922 were selected to quantify the hinge bending motion as they lie on opposite sites of the catalytic cleft and their distance is directly related to the opening and closing of the active site. In contrast to the hinge bending motion, which occurs in two dimensions (linear), the twisting motion occurs in three dimensions. For this motion, the highest correlated functional quantity was the RMSD of the C*α* of the active site residues (*RMSD_act_*), which is a non-linear metric.

For *d_LM_*, the collective vector *α* was optimized by maximizing the Pearson's correlation coefficient (R), yielding linear models for the WT and mutant *d_LM_*. We used the first 35 ns of the production run for model building and the rest 15 ns for cross-validation (Figure S15 C–H). To avoid over-fitting of the model in the selection of the basis set, the Pearson's correlation coefficients of the model-building (*Rm*) and the cross-validation set (*Rc*) were plotted as a function of the number of eigenvectors used as a basis set (Figures S15 A and S15 B). The hinge bending motion between the two lobes of the kinase domain from the WT and H1047R p110α is illustrated in Figures S15A, B and video S3. Our analysis shows that the P-loop is closer to the catalytic loop in the WT than in the mutant throughout the course of the motion (video S3).

**Figure 7:**
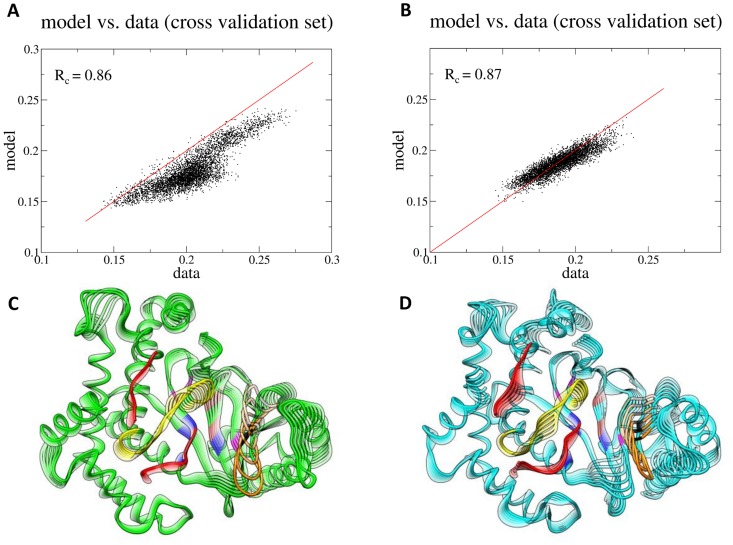
Functional mode analysis of the Cα RMSD of active site residues. (A/B) Scatter plots of the data versus the model using the cross-validation sets only. (C/D) The functional mode representing the kinase C- and N-lobe twisting motion for the WT and mutant proteins, respectively. Red: activation loop; yellow: catalytic loop; orange: P-loop; brown: hinge; magenta: adenine pocket; blue: affinity pocket (hydrophobic region I); black: specificity pocket.

For the description of the twisting motion, the RMSD*_act_* was optimized by maximizing the mutual information (MI) coefficient (see Supporting Information Text S1, section A6 for more details). The MI is used to quantify non-linear, higher order correlation. We used the first 40 ns of the production phase for model building the last 10 ns for cross-validation (Figures S16 A–D). For the optimization of the non-linear model with the MI, we used less than 20 eigenvectors to avoid over-fitting. As shown in Figures S16 A, B, the difference between *R_m_* and *R_c_* reaches a minimum when the number of used eigenvectors is 17 in the WT and 13 in the mutant. The two basis sets yielded a Pearson's correlation values of 0.86 and 0.87 for the WT and the mutant trajectory, respectively (Figures 7 A, B), which denote high correlation between the RMSD*_act_* and the twisting motion of the kinase lobes. In both the mutant and the WT, the P-loop lies on the same plane, however, as the motion progresses, it is shifted outwards in the mutant with respect to the WT, broadening the solvent accessibility of the mutant catalytic cleft (Figures 7 C, D and video S4). A more open conformation of the P-loop in the mutant structure was also observed through our polar contact analysis within the active site of the WT and mutant proteins (see above). A greater catalytic cleft may lead to enhanced substrate accessibility.

Moreover, the catalytic loop of the WT comes closer to the ATP binding site thus reducing the volume of the pocket. The mutant activation loop lies below the activation loop of the WT in the starting position, but this positioning is reversed in the final position. The average conformations of the kinase domains show that the P-loop of the WT p110α curls inwards, towards the ATP-binding cavity when compared with the H1047R p110α, which results to a greater catalytic cleft in the mutant protein (Figure S17).

Moreover, in order to quantify the overlap between the mutant and WT trajectory eigenspaces, we calculated the Root Mean Square Inner Product (RMSIP) for all corresponding eigenvectors arising from PCA for the kinase domain (see Text S1, section A5 and Table S7). The mutant and the WT trajectory RMSIP yielded a normalized value of 0.23±0.01 for the kinase domain PCA and for the five independent trajectories, indicating that the eigenspaces of the WT and the mutant are different. In comparison, the RMSIP of the WT trajectories is 0.38±0.03 and for the mutant RMSIP = 0.42±0.01. In other words, the motions along each PC of the WT did not correspond to the motions of the equivalent principal component of the mutant as shown by their overlap, which was 23%.

## Discussion

**Figure 8:**
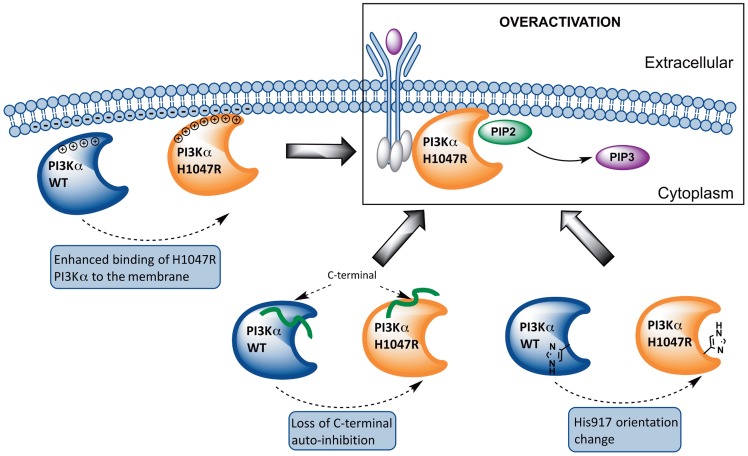
Proposed model of the overactivation mechanism of the *PIK3CA* mutant H1047R based on structural and dynamic differences with its WT counterpart. From left to right: The mutant protein accumulates positive charge in regions that contact the cell membrane and displays higher membrane binding affinity compared to the WT protein. The auto-inhibitory role of the C-terminal tail, which strictly controls the DRH motif to limit its access to the catalytic site, is abolished in the mutant protein due to loss of crucial intermolecular interactions. In the WT protein, His-917 of the DRH motif, points away from the active site thus preventing ATP hydrolysis more efficiently, while in the mutant PI3Kα structure, His917 points towards the active site, a conformation that is also observed in the structure of the active PI3Kγ.

Results presented herein lead to a model of the overactivation mechanism of the commonly-expressed *PIK3CA* mutant H1047R based on structural and dynamic differences with its WT counterpart, schematized in Figure 8. SPR experiments show that the H1047R mutant binds liposomal membranes with higher competence. This finding is rationalized through MD simulations and subsequent electrostatic potential calculations, which verify that the mutant protein accumulates positive charge on the membrane binding domains of *PIK3CA*. This accumulation of positive charge explains the experimental finding that the mutant binds membranes rich in anionic lipids with higher capacity than the WT. Previous studies have shown that the C-terminal tail of the mutant is more solvent exposed than its WT counterpart, which is also confirmed through our MD simulations. Moreover, we verify the prediction of a second, unexpected binding pocket close to the area of the mutation, recently discovered by X-ray crystallography. Following the agreement with experimental results, we extend our studies to highlight the series of events that lead to the overactivation of this protein kinase mutant. Our results support an auto-inhibitory role of the C-terminal tail in the WT protein, which strictly controls the DRH motif to limit its access to the catalytic site.

We propose that the weakening of this role in the H1047R mutant through loss of crucial intermolecular interactions is a plausible explanation of the elevated kinase activity of the enzyme. One major difference between the polar contact network of the WT and H1047R is the loss of the hydrogen bond connecting Arg-949 and catalytic Asp-915 of the DRH motif in the mutant, which occurs in the WT in 78±6% occurrence, while it is absent in the mutant. Arg-949 is known for conferring specificity to PIP2 in PI3Kα [8]. The same functional role has also been reported in the γ isoform [21]. The difference in the polar contacts, position, and consequently the availability of the positively charged residue, Arg-949 (along with Lys-948 and Arg-951), alters the configuration of the activation loop and exposes its positive charges, making it seemingly more capable to bind to the membrane and accommodate negatively charged phosphoinositide headgroups. We also suggest that the abrogation of the Arg-949 - Asp-915 interaction in the mutant may contribute to the overactivity of the enzyme, since when these residues are left unrestrained they have enhanced access to the catalytic site [8].

In the H1047R p110α, Arg-1047 disrupts the last helical turn of kα11 and unwinds the hairpin observed in the crystal structure (compare Figures 3 A and 3 B) [12]. One plausible explanation is that substitution of the bulky imidazole ring by the longer aliphatic straight chain capped with the positively charged guanidium group, rules out the hydrogen bond interaction with the Leu-956 backbone amine, which is conserved throughout the trajectory of the WT, but is completely absent in the mutant (Figures 2, S5 and S6, Table S3). Mandelker et al. [12] also accentuates the loss of this hydrogen bond in the crystal structures of H1047R p110α/p85α-niSH2 complex (PDB codes 3HIZ, 3HHM) and suggests that it is this interaction that stabilizes the WT activation loop. According to our data, the activation loop in the mutant structure is more flexible than in the WT and this can partially explain the oncogenic phenotype of the H1047R p110α (Table S6). On the other hand, the loss of interaction between residues 1047 and 956, evoke the disruption of the simultaneously occurring hydrogen bond between Leu-956 backbone carbonyl and Thr-1053 side chain carboxyl. These two stable hydrogen bonds, mediated by Leu-956 in the WT, preserve the hairpin formation and hold the C-terminal tail in an arrangement that favors interaction with the DRH motif of the catalytic loop. This interaction with the DRH motif in the WT keeps His-917, which participates in ATP hydrolysis, pointing away from the active site thus preventing ATP hydrolysis more efficiently. This conformation of His-917 has also been observed in the inactive form of Vsp34 (the primordial PI3Kα), where the catalytic His-807 points away from the ATP site (see Figures 2 C and 3 of Ref. [15]). In contrast, in the mutant PI3Kα structure, His917 points towards the active site, a conformation that is also observed in the structure of the active PI3Kγ, where His-745 is the residue participating in catalysis [15].

Additionally, the C-terminal tail of the H1047R mutant is significantly more solvent exposed in the H1047R mutant compared to the WT. It is worth noting that Hon et al. [8] reported that all mutations in the kα11/kα12 elbow (H1047L, H1047R, G1049R) exhibited a few fold higher levels of hydrophobic binding to neutral lipids and electrostatic binding to negatively charged lipids than the WT p110α, which may be associated with enhanced solvent accessibility of the mutant kα12 region and in particular higher solvent accessible area per residue, as indicated by Figure S9.

The importance of the final C-terminal helices of the p110*α* catalytic subunit in the regulation of the enzyme has been previously highlighted [14]–[16]. This regulatory arch encircles the catalytic and activation loops and is believed to control the enzymatic activity [14]. The last helix, kα12, which is disordered in p110α and p110δ, has two additional roles: (a) an activating role when in contact with the membrane [8], (b) an auto-inhibitory role when the enzyme is not interacting with the membrane [14]–[16]. In the latter case, kα12 locks the catalytic loop in an inactive state, presumably by shielding the conserved catalytic DRH motif (915–917) from performing futile ATP hydrolysis. This second role has been inferred from inspection of the crystal structures of PI3K isoforms α, β, γ, δ and their paralogue in Drosophila melanogaster Vps34, as well as from truncation of the C-terminus of p110α, p110β and Vps34 that resulted to enhanced basal ATPase activity in the absence of lipid substrate [8], [15], [16]. These results verify the self-inhibitory role of the C-terminus. Remarkably, the C-terminus interaction with the activation loop is relieved in the H1047R p110α (Tables S8, S9, S10), which may well be part of the explanation of the enhanced kinase activity of that enzyme.

The highly conserved DFG motif (933–935) at the beginning of the activation loop is believed to adopt different configurations during the various steps of the catalytic cycle of kinases [22] In PI3K, the aspartate side chain of DFG (Asp-933) bends in order to form polar contacts with the last phosphate group of ATP [23]. This is believed to be the “in” conformation which designates the active state, whereas in the “out” conformation the aspartate side chain extends straight towards the ribose ring. Our results show that both WT and mutant Asp-933 adopt an “in-like” conformation, albeit Asp-933 of the WT structure is frequently turned away from the ATP binding site due to a high-frequency hydrogen bond with Arg-916 and a low frequency hydrogen bond with the Gly-935 backbone amine (Table S3 and videos S1 and S2). Both these polar contacts have significantly lower frequencies in the H1047R p110α and thus the mutant Asp-933 shows a tendency to assume an “in-like” conformation more frequently than the WT, providing an advantage to the former regarding ATP binding. Moreover, Asp-933 in both protein structures forms a salt- bridge with residue Lys-802 of the active site. The equivalent of residue Lys-802 in PI3Kγ crystal structure (Lys-833) forms a hydrogen bond with the pan-PI3K inhibitor PIK-90 [24]. It is, therefore, plausible that Lys-802 plays a crucial role in the design of inhibitors like PIK-90. Lastly, in accordance with a previous simulation [19], the change in the orientation of Asp-933 is accompanied by a flip of the Phe-934 side chain that renders it more exposed to substrates entering the ATP-binding site. These observations may provide an important basis for the design of mutant selective inhibitors.

Changes in the polar contact network within the active site were also observed. We discern a change in the hydrogen bonding frequency between the side chain of Asp-810 and the backbone of Phe-934 from the DFG motif. This hydrogen bond loss could be exploited in the design of mutant-specific inhibitors targeting Asp-810, given that ligand binding in the WT active site would have to overcome the additional enthalpic cost for breaking these two frequent hydrogen bonds. Moreover, the specificity pocket residue Trp-780 is involved in two hydrogen bonds with Arg-770 with much higher frequency in the mutant than in the WT protein, providing valuable information for the design of inhibitors targeting the H1047R p110α.

Finally, through FMA we show that differences in the twisting motion of the kinase lobes exist, with the mutant having a greater opening of the catalytic cleft, which may favor ATP binding and thus influence kinase activity. Changes in the twisting motion have been previously proposed to alter kinase activity [20]. Through the FMA and polar contact analysis, we observe a wider conformation of the P-loop relative to the ATP pocket in the mutant structure, while at the same time it is more compact compared to the WT structure, which could lead to enhanced accessibility of the active site.

Overall, understanding how the H1047R mutation causes the enhanced activity of the protein in atomic detail is of paramount importance for developing mutant-specific therapeutics for cancer.

## Materials and Methods

### Model construction and refinement

A full description of the methods can be found in the Text S1 (sections A1–A6). Briefly, two models of the WT p110α were constructed: in Model 1a the missing C2 domain loop residues 415–423 were created through loop modeling (Figure S18 B, Text S1, section A2). In Model 1b, residues 335–361 and 401–428 were re- constructed through homology modeling (Text S1, section A1 and Figure S18 C), using as a template the solution NMR structure of the human C2 domain with PDB accession code 2ENQ (Figure S18 D), due to the low electron density of the WT p110α structure (2RD0) at this area (Figure S19). The rest of the missing loops of 2RD0 were created through loop modeling and share the same coordinates in both Models 1a and 1b. Model 2 was built from the PDB structure 3HIZ [12]. C2 domain residues 335–361 and 402–428 were rebuilt from structure 2ENQ and residues 857–884 were rebuilt using PDB structure 3HHM as a template through homology modeling due to low electron density in this region (Text S1, section A3).

### Molecular dynamics simulations and functional mode analysis

The MD trajectories for the WT and H1047R p110α were generated with NAMD v2.7 [25], using the CHARMM22 all-atom force field with the CMAP correction [26], [27] and the TIP3P water model [28]. Five independent simulations for each protein were performed. Moreover, for the WT protein, we simulated both Model 1a and one of Model 1b in order to assess the effect of the remodeled C2 domain. All proteins were solvated into a cubic box large enough to ensure a 30 Å minimum separation of the protein from its periodic images. Na+ counter-ions were placed randomly in the system to neutralize the total charge (see Text S1, section A4 for more details). The production runs were performed under constant pressure, temperature, and number of particles (NPT). The convergence of our simulations was evaluated using the total Cα carbon RMSD, while to ensure that each set of independent simulations corresponds to the same conformational protein phase space, we plotted the 2 d projection of the trajectories on the first two eigenvectors of each simulation (Text S1, section A5). The trajectory was analyzed with nMOLDYNv3.0.8 [29], MMTK-2.7.4 [30], GROMACS tools v4.5.5 [31], NAMD v2.7 [25], [32], PDB2PQR [32], APBS [33], and VMD [34]. Functional Mode Analysis (FMA) was performed as discussed in Ref. [35]. Binding site prediction was performed with the QSiteFinder web server [17]. For more details on trajectory analysis see the Text S1, section A5.

### Surface Plasmon Resonance (SPR)

The ProteOn biosensor (Bio-rad) was used for SPR analysis. ProteOn uses a unique 6×6 chip array (positions L1 to L6 are vertical and positions A1 to A6 are horizontal) that allows running experiments in a grid format. The LCP memLayer kit (Bio-Rad) was used to tether liposomes (in two consecutive layers) on the chip up to ∼3500 RU, using the vertical (L) channel direction. Blank control channels (L direction) that were treated equivalently to liposome loaded channels but lacked liposomes, were used for data reference. Appropriate concentrations of WT and H1047R PI3K (diluted in SPR running buffer just before run) were injected over the chip using the parallel (A) channels. To ensure high experimental uniformity and confidence in SPR data comparisons, each binding experiment was performed in “one shot”, i.e. both WT and H1047R were injected in the same parallel injection (occupying different A positions). Background PI3K binding and bulk effects within each injection were referenced using blank L channels. Experiments were repeated at least 3 times, using fresh liposomes loadings. SPR running buffer: 10 mM NaPi pH 7.4, 150 mM NaCl, 0.1 mg/ml BSA.

### Proteins

Pure human WT p110α/p85α and mutant p110α(H1047R)/p85α heterodimers were purchased from Millipore. Protein activity was verified using PI3-Kinase HTRF Assay (Millipore). Proteins in 50 mM Tris/HCl pH 8.0, 300 mM NaCl, 0.1 mM EGTA, 0.03% Brij-35, 270 mM sucrose, 0.2 mM PMSF, 1 mM benzamidine, 0.1% 2-mercaptoethanol were aliquoted and stored at −80°C before use.

### Liposomes

Liposomes have been prepared using lipids isolated from the cancer cell line HCT116, which carries a mutation in exon 20 of *PIK3CA* (H1047R) [2], according to Folch's method [36]. Then, L-α-phosphatidylinositol-4,5-bisphosphate (brain, porcine, ammonium salt, Avanti Polar Lipids, Inc.) was added to the extracted lipids (PIP2 concentration was 2% of the total lipids), and the mixture was immediately dried under N_2_ stream. When the sample was completely dried, lipids were left for another 30 minutes under the N_2_ stream, followed by speed-vacuum for 1 hr. Subsequently, water was added to the dried lipids and the mixture was incubated at room temperature for 1 hr, while it was vortexed every 10 minutes. The liposomal preparation was subjected to 5 freeze/thaw cycles and was sonicated in a waterbath for 30 minutes. Finally, the liposomes were extruded using the Avanti mini-Extruder apparatus, according to manufacturer's instructions, in order to obtain a homogeneous preparation of unilamelar liposomal vesicles at a size of 100 nm.

## Supporting Information

Figure S1 **The models of the full-length (A) WT p110α, (B) H1047R mutant.** Regions created with loop modeling are colored red. In blue are shown the regions reconstructed through homology modeling. The indices of the modeled residues are indicated. His-1047 and Arg-1047 are depicted with stick representation in magenta in (A) and (B), respectively.
(TIF)

Figure S2 **RMSD of the Cα carbons of p110α.** (**A**) WT, (**B**) H1047R p110α. The highly flexible loop residues 1–7, 231–240, 291–330, 410–417, 505–530, 863–872, 941–952, 1047–1068 have been excluded from the calculation.
(TIF)

Figure S3 **2D projection of the trajectories on the first two eigenvectors of each independent simulation** for the WT and the H1047R mutant proteins.
(TIF)

Figure S4 **The structure of the L-histidine showing the π (or δ) and τ (or ε) imidazole nitrogen atoms.**
(TIF)

Figure S5 **Snapshots close to the mutation site of the first cluster representatives of the five WT p110α simulations.** The activation loop (res. numbers 933–958) is colored orange and the catalytic loop (res. numbers 909–920) is colored yellow. Residues forming hydrogen bonds are shown in licorice representation.
(TIF)

Figure S6 **Snapshots close to the mutation site of the first cluster representatives of the five 1047R p110α simulations.** The activation loop (res. numbers 933–958) is colored orange and the catalytic loop (res. numbers 909–920) is colored yellow. Residues forming hydrogen bonds are shown in licorice representation.
(TIF)

Figure S7 **Stacking of the WIF motif for the WT protein** as indicated by the center of mass distance between tryptophan, phenylalanine ring, and the side chain of isoleucine for the five simulations of the WT protein.
(TIF)

Figure S8 **Stacking of the WIF motif for the H1047R protein** as indicated by the center of mass distance between tryptophan, phenylalanine ring, and the side chain of isoleucine for the five simulations of the H1047R mutant protein.
(TIF)

Figure S9 **Average solvent accessible area and standard deviation of the C-terminal residues 1032–1068** from five independent unbiased MD simulations for the WT and H1047R mutant proteins. Residues Met-1043, Trp-1057 as well as the mutated residue 1047 have higher solvent accessible area in the mutant, whereas Met-1055 and Asp-1056 are more solvent accessible in the WT protein.
(TIF)

Figure S10 **Snapshots close to the active site of the first cluster representatives of the five WT p110α simulations.** The P-loop (res. numbers 771–777) is shown in yellow, the affinity pocket (res. numbers 810, 836, 848, 932) in pink, the adenine pocket (res. numbers 800, 836, 922, 930) in orange, and the hinge region (res. numbers 849, 851) is colored in purple. Residues forming hydrogen bonds are shown in licorice representation.
(TIF)

Figure S11 **Snapshots close to the active site of the first cluster representatives of the five 1047R p110α simulations.** The P-loop (res. numbers 771–777) is shown in yellow, the affinity pocket (res. numbers 810, 836, 848, 932) in pink, the adenine pocket (res. numbers 800, 836, 922, 930) in orange, and the hinge region (res. numbers 849, 851) is colored in purple. Residues forming hydrogen bonds are shown in licorice representation.
(TIF)

Figure S12 **Average solvent accessible area and standard deviation of the active site residues** from five independent unbiased MD simulations for the WT and H1047R mutant proteins. Following Williams' notation (Williams et al., 2009), the catalytic site was defined to consist of the P-loop (771–777), the hinge region (849, 851), the adenine pocket (800, 836, 922, 930), the gatekeeper (848), the affinity pocket (810, 836, 848, 932) and the specificity pocket (772, 780).
(TIF)

Figure S13 **The electrostatic potential on the surface of the average conformation of the WT and H1047R p110α.** The images depict membrane interaction areas of the WT (A, C) and H1047R mutant (B, D) cluster 2 and cluster 3 representatives. The surface has been colored with a color scale from −7 eV to 7 eV, with red representing negative charge, white neutral and blue positive. The charge value corresponds to the solvent accessible surface of the protein, namely 1.4 Å far from the surface. These structures were derived from a cluster analysis including all the Cα carbon with a cutoff of 1.7 Å.
(TIF)

Figure S14 **The kinase domain of the same model (cyan) overlaid onto the WT model (green).** Residues created through loop modeling are colored orange in the mutant and red in the WT. The initial configuration of the C-termini of the two proteins occupy the same area in space. Residues constructed through homology modeling in the mutant are blue. Arg-1047 and His-1047 are depicted as magenta sticks.
(TIF)

Figure S15 **Functional mode analysis of the distance between the Cα carbons of Leu781 and Met922 (d_LM_).** (A/B) the functional mode representing the kinase C- and N-lobe hinge bending motion for the WT and mutant proteins, respectively. Red: activation loop; yellow: catalytic loop; orange: P-loop; brown: hinge; magenta: adenine pocket; blue: affinity pocket (hydrophobic region I); black: specificity pocket. (C/D) the functional quantity f (d_LM_) versus the simulation time (black curve) for the WT and mutant, respectively. Red and green curves represent the model in the model and cross-validation building set, respectively. (E/F) scatter plots of the data versus the model using the cross-validation sets only. (G/H) Correlations R_m_ and R_c_ for d_LM_ as a functions of the number of eigenvectors used during the optimization; blue arrows indicate the number of eigenvectors used for the basis set.
(TIF)

Figure S16 **Functional mode analysis of the Cα RMSD of active site residues.** (A/B) Correlations R_m_ and R_c_ for RMSD as a functions of the number of eigenvectors used during the optimization; blue arrows indicate the number of eigenvectors used for the basis set. (C/D) the functional quantity f (RMSD) in nm versus the simulation time (black curve) for the WT and mutant, respectively. Red and green curves represent the model in the model and cross-validation building set, respectively.
(TIF)

Figure S17 **Average conformation of the ATP-binding site of the WT (green) and the H1047R (cyan) p110α.** The P-loop of the WT curls inward towards the ATP-binding cavity when compared with the H1047R p110α, so than the active site of the WT is more closed than the mutant. Red: activation loop; yellow: catalytic loop. In the final state, the Cα atoms of the two P-loops are within an average distance of 4.79 Å. The ATP is placed manually to indicate the active site.
(TIF)

Figure S18 **The C2 domain of the human WT p110α.** The two ends of the missing loop (positions 414 and 424) are colored yellow. (**A**) The C2 domain of the WT p110α crystal structure (PDB accession code: 2RD0). (**B**) The C2 domain of the same structure after loop modeling. The predicted conformation of the loop residues (415–423) is shown in red. (**C**) The C2 domain of the same structure after homology modeling using the solution NMR structure of the isolated human C2 domain as a template (PDB accession code: 2ENQ). Blue indicates the part that was re-modeled. (**D**) The solution NMR structure of the isolated human C2 domain (PDB accession code: 2ENQ).
(TIF)

Figure S19 **Remodeling of the C2 domain.** (**A**) Electron density around the C2 domain in the WT p110a (PDB code 2RD0). Yellow sticks represent residues that were remodeled. (**B**) The Cα RMSD with respect to the starting conformation of the original C2 domain (black) versus the remodeled one (red). The remodeled C2 domain converges faster to average RMSD value, meaning that it is closer to equilibrium than the C2 domain in the original crystal structure.
(TIF)

Table S1 **List of simulated systems.** The analysis of the trajectories was performed in the last 50 ns of each trajectory.
(DOCX)

Table S2 **Population (in frames) of the first three clusters of the whole p110α subunit (cutoff = 1.7 Å) and the kinase domain (residues 697–1068) (cutoff = 1 Å) for Simulation 1 of the mutant and WT proteins.** Although the total population of the first three clusters is comparable, the majority of the frames in the mutant are accumulated in the first cluster, indicating that the H1047R p110α protein visits less conformational states than the WT p110α during production run. The first three clusters include almost the same number of frames and cover the majority of the population (94.1% and 91.6% for the WT versus 92.6% and 90.7% for the mutant).
(DOCX)

Table S3 **Important hydrogen bonds and their frequencies in the WT and mutant p110α kinase domain.** The hydrogen bonds are sorted by the amino acid in the donor-acceptor pair with the lowest index. Atom names follow the CHARMM force field naming scheme.
(DOCX)

Table S4 **Average distance and standard deviation in Å from five independent unbiased MD simulations.**
(DOCX)

Table S5 **Average area and standard deviation of the C-terminal and catalytic site residues from five independent unbiased MD simulations.**
(DOCX)

Table S6 **Average RMSF values and standard errors (Å) of the functionally important loops of the kinase domain in the WT and H1047R p110α.**
(DOCX)

Table S7 **Overlap of the covariance matrices between the independent runs for both WT and mutant calculated by means of the Root Mean Square Inner Product (RMSIP).**
(DOCX)

Table S8 **Hydrogen bond frequencies within the C-terminal tail (res. numbers 1048–1068) and the activation loop (res. numbers 933–958) of the WT p110α protein.** The hydrogen bonds between the two domains are shown in bold.
(DOCX)

Table S9 **Hydrogen bond frequencies within the C-terminal tail (res. numbers 1048–1068) and the activation loop (res. numbers 933–958) of the H1047R mutant p110α protein.** The hydrogen bonds between the two domains are shown in bold.
(DOCX)

Table S10 **Salt-bridge frequencies in the WT and mutant p110α kinase domain.** The salt-bridges are sorted by the amino acid in the donor-acceptor pair with the lowest index.
(DOCX)

Text S1 **The supporting information text provides details on the model construction and refinement.**
(DOCX)

Video S1 **Stabilizing interaction at the PIP2 binding site in the WT p110α.** Residues that participate in important polar contacts are depicted as sticks. The C-terminus is colored green, the activation loop red, the catalytic loop yellow and the WIF (1057–1059) motif purple.
(MP4)

Video S2 **Stabilizing interaction at the PIP2 binding site in the H1047R p110α.** Residues that participate in important polar contacts are depicted as sticks. The C-terminus is colored green, the activation loop red, the catalytic loop yellow and the WIF (1057–1059) motif purple.
(MP4)

Video S3 **The hinge bending motion of the kinase domain.** WT p110α is colored green and H1047R cyan.
(MP4)

Video S4 **The twisting motion between the N- and C-lobe of the kinase domain.** WT p110α is colored green and H1047R cyan.
(MP4)
