# Supplementary figures and images for: Investigating the Structure and Dynamics of the PIK3CA Wild-Type and H1047R Oncogenic Mutant
Source: PLoS Comput Biol. 2014 Oct 23;10(10):e1003895. doi: 10.1371/journal.pcbi.1003895 (PMC4207468; doi:10.1371/journal.pcbi.1003895)

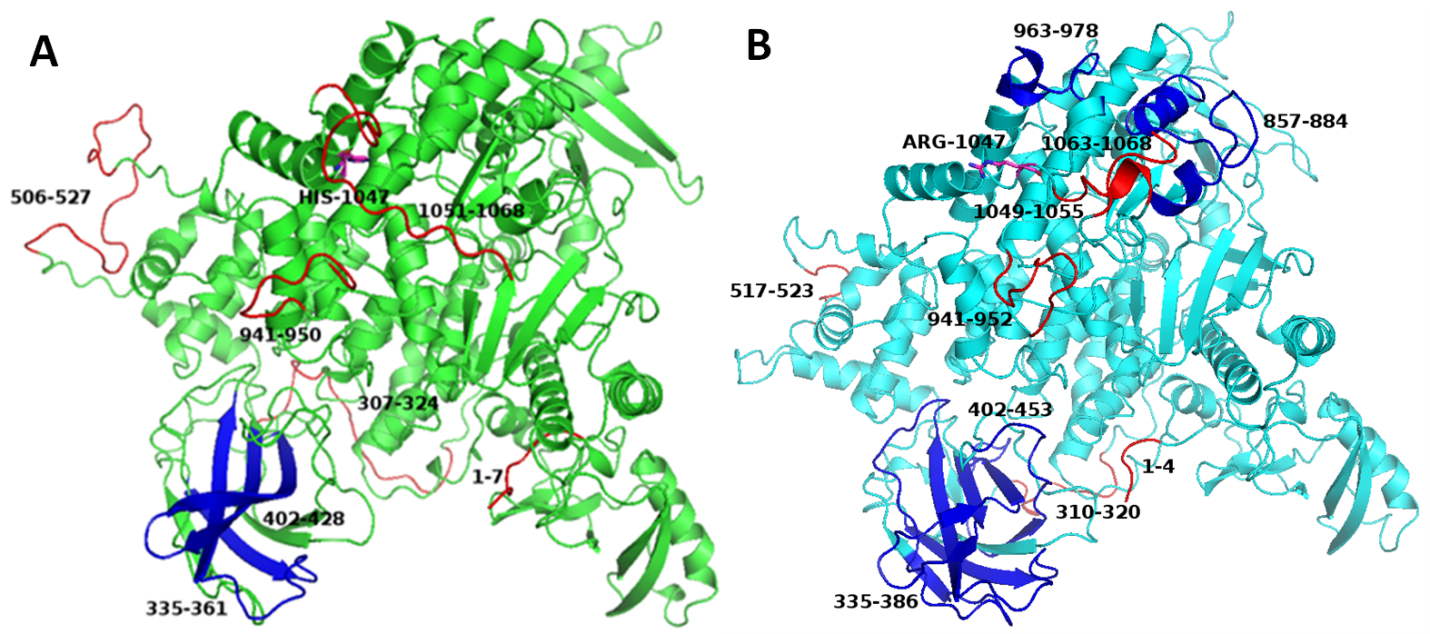

Supplement: Figure S1 — The models of the full-length (A) WT p110α, (B) H1047R mutant. Regions created with loop modeling are colored red. In blue are shown the regions reconstructed through homology modeling. The indices of the modeled residues are indicated. His-1047 and Arg-1047 are depicted with stick representation in magenta in (A) and (B), respectively. (TIF) [file pcbi.1003895.s001.tif]

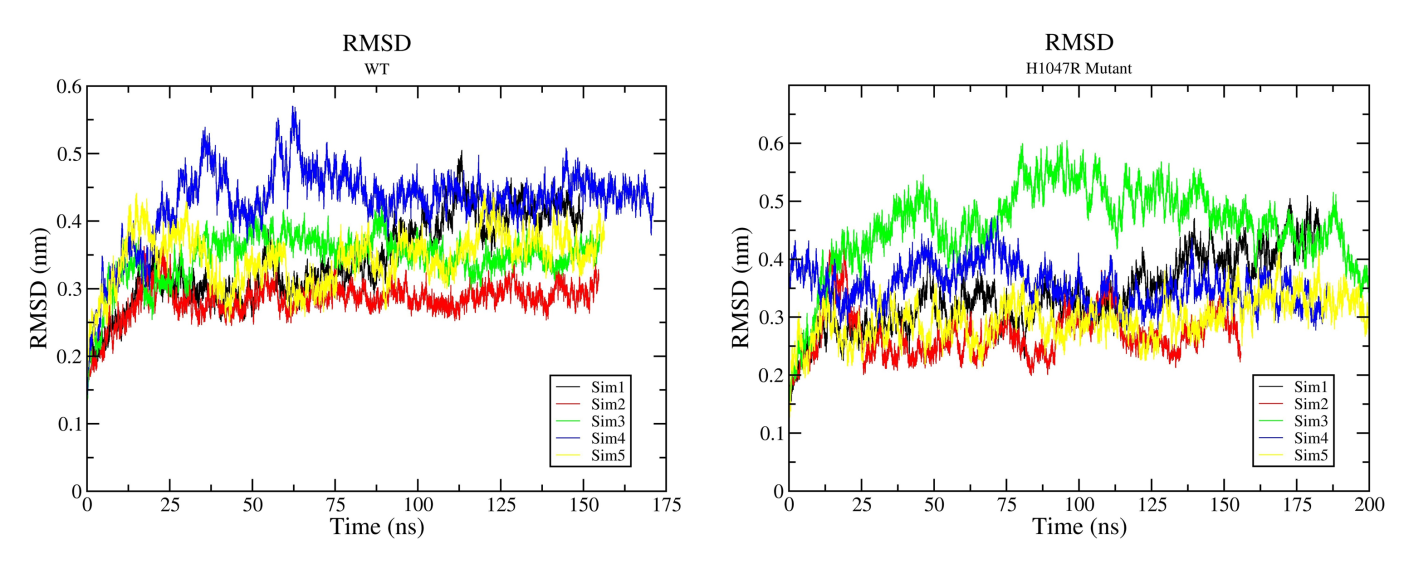

Supplement: Figure S2 — RMSD of the Cα carbons of p110α. (A) WT, (B) H1047R p110α. The highly flexible loop residues 1–7, 231–240, 291–330, 410–417, 505–530, 863–872, 941–952, 1047–1068 have been excluded from the calculation. (TIF) [file pcbi.1003895.s002.tif]

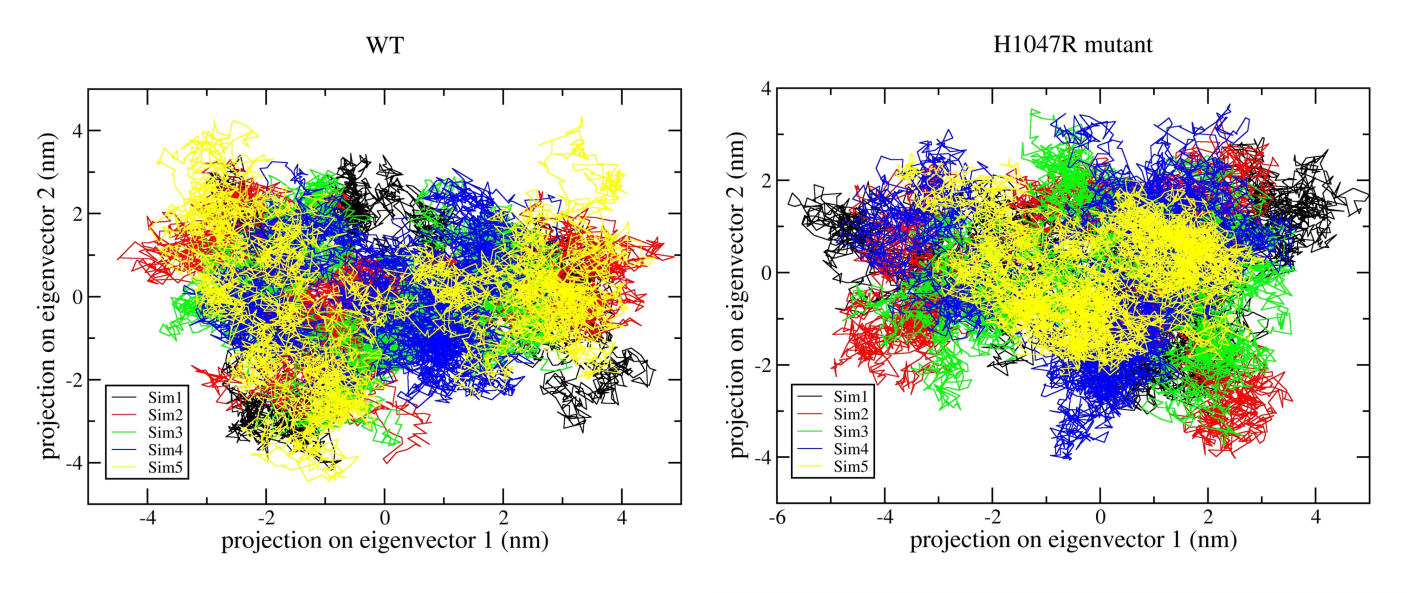

Supplement: Figure S3 — 2D projection of the trajectories on the first two eigenvectors of each independent simulation for the WT and the H1047R mutant proteins. (TIF) [file pcbi.1003895.s003.tif]

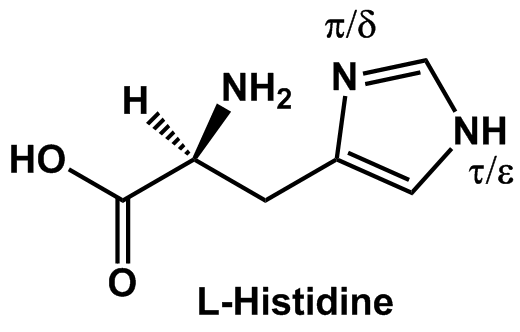

Supplement: Figure S4 — The structure of the L-histidine showing the π (or δ) and τ (or ε) imidazole nitrogen atoms. (TIF) [file pcbi.1003895.s004.tif]

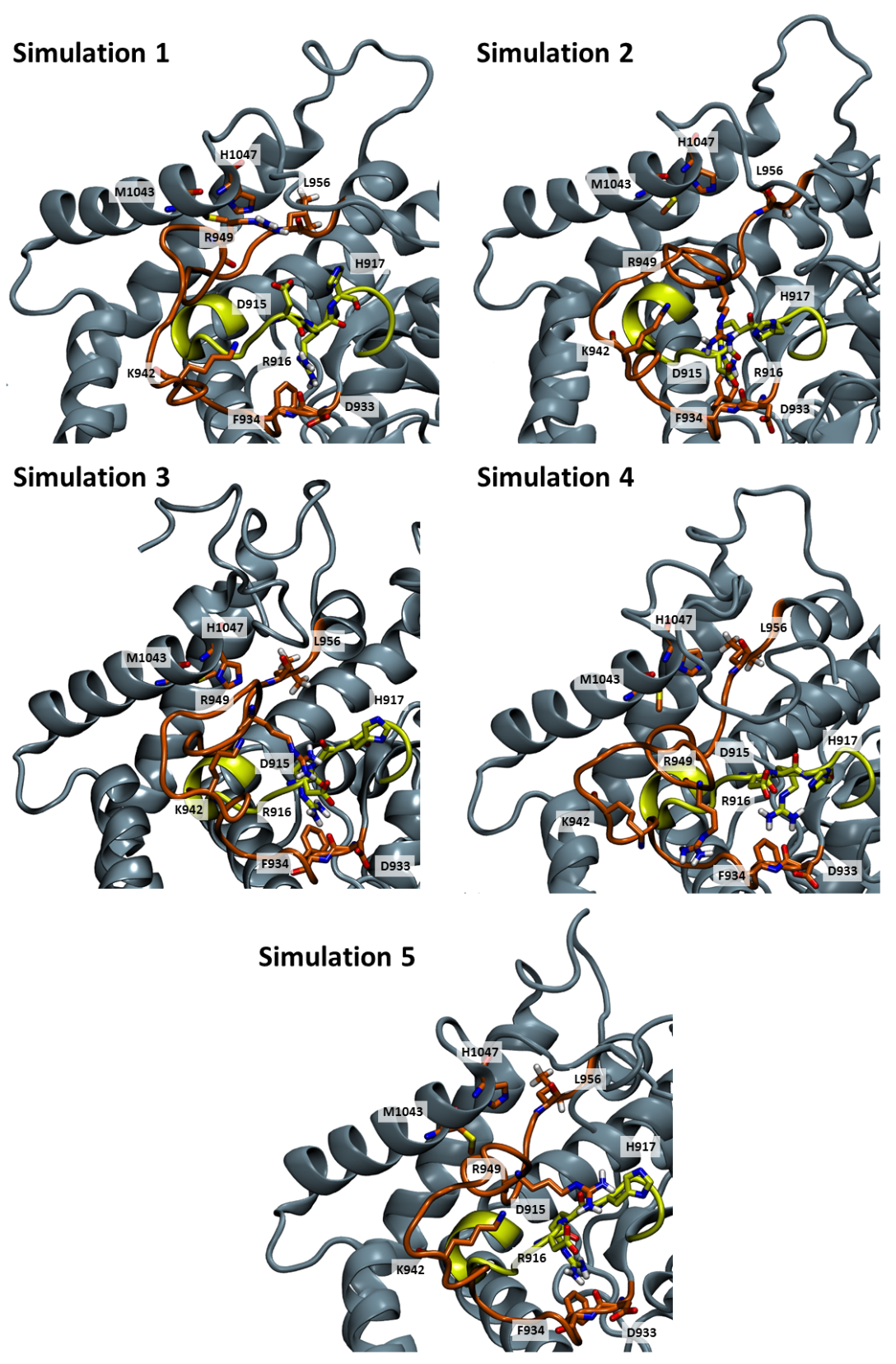

Supplement: Figure S5 — Snapshots close to the mutation site of the first cluster representatives of the five WT p110α simulations. The activation loop (res. numbers 933–958) is colored orange and the catalytic loop (res. numbers 909–920) is colored yellow. Residues forming hydrogen bonds are shown in licorice representation. (TIF) [file pcbi.1003895.s005.tif]

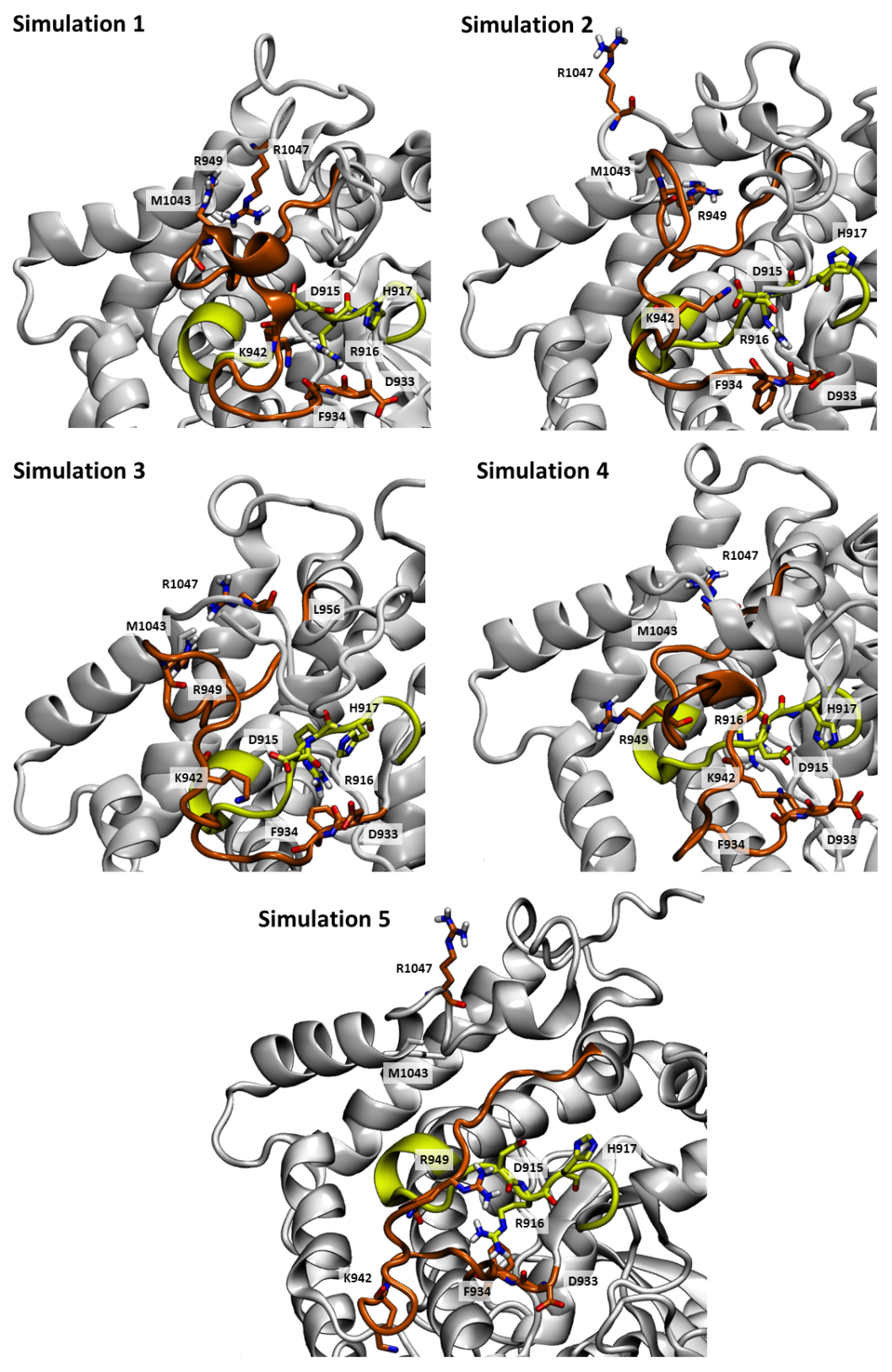

Supplement: Figure S6 — Snapshots close to the mutation site of the first cluster representatives of the five 1047R p110α simulations. The activation loop (res. numbers 933–958) is colored orange and the catalytic loop (res. numbers 909–920) is colored yellow. Residues forming hydrogen bonds are shown in licorice representation. (TIF) [file pcbi.1003895.s006.tif]

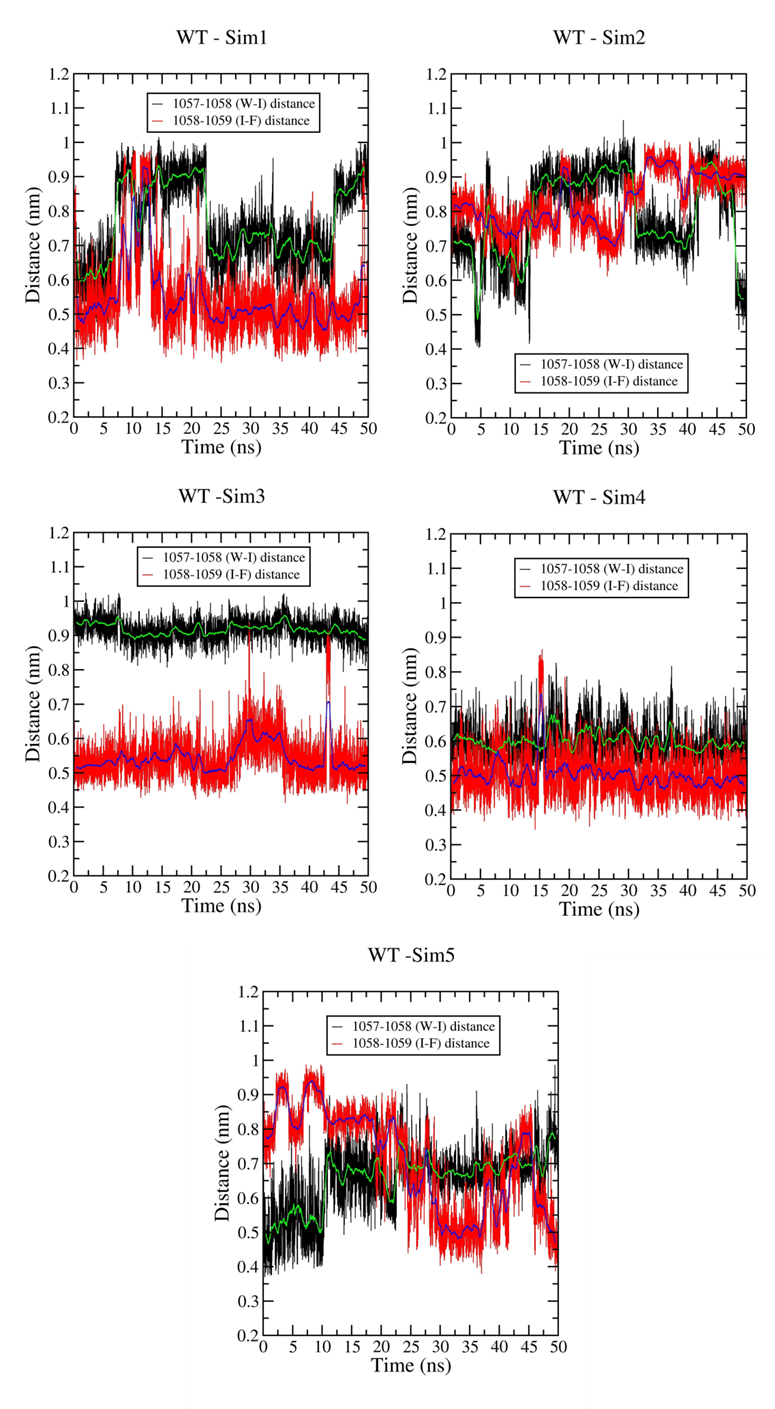

Supplement: Figure S7 — Stacking of the WIF motif for the WT protein as indicated by the center of mass distance between tryptophan, phenylalanine ring, and the side chain of isoleucine for the five simulations of the WT protein. (TIF) [file pcbi.1003895.s007.tif]

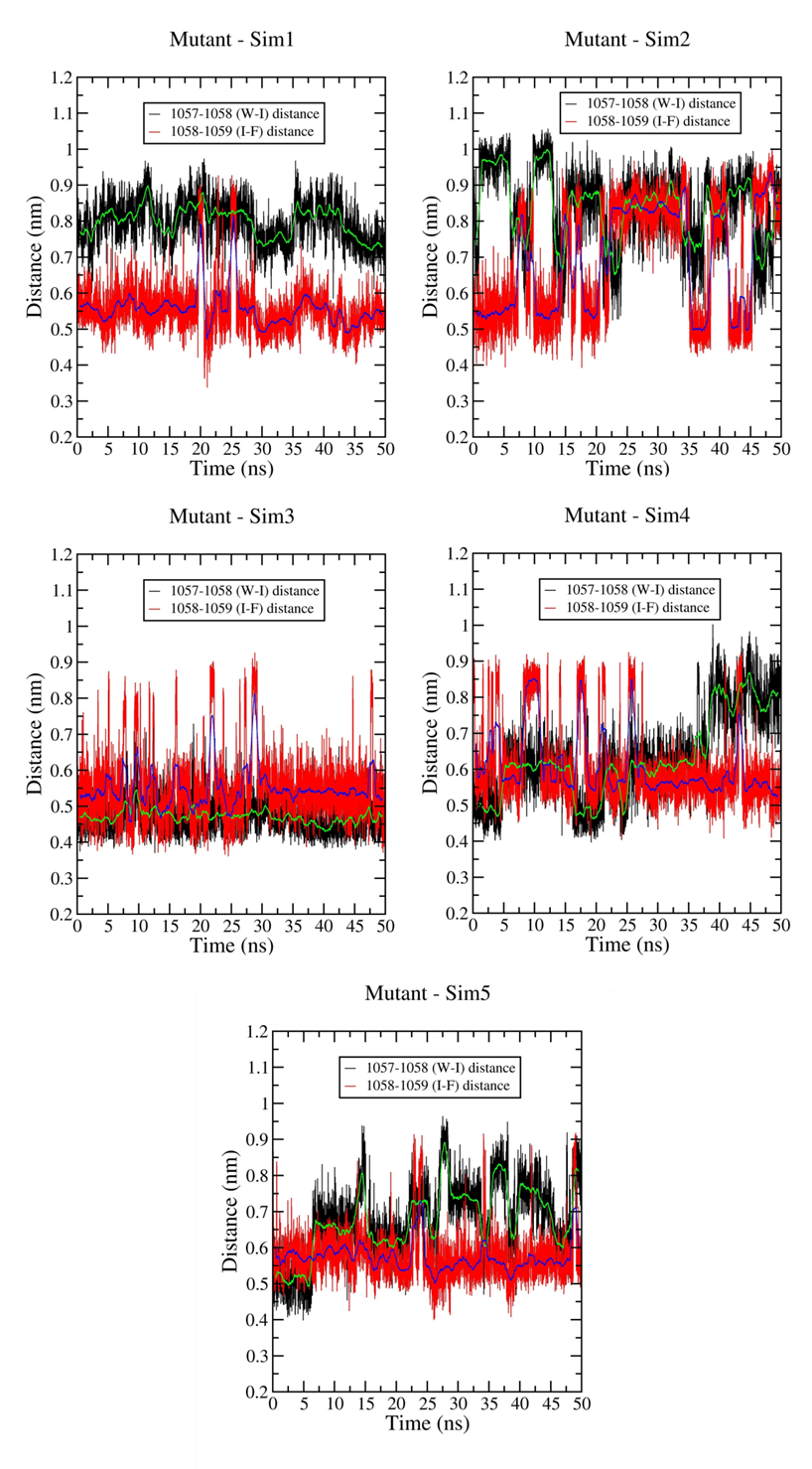

Supplement: Figure S8 — Stacking of the WIF motif for the H1047R protein as indicated by the center of mass distance between tryptophan, phenylalanine ring, and the side chain of isoleucine for the five simulations of the H1047R mutant protein. (TIF) [file pcbi.1003895.s008.tif]

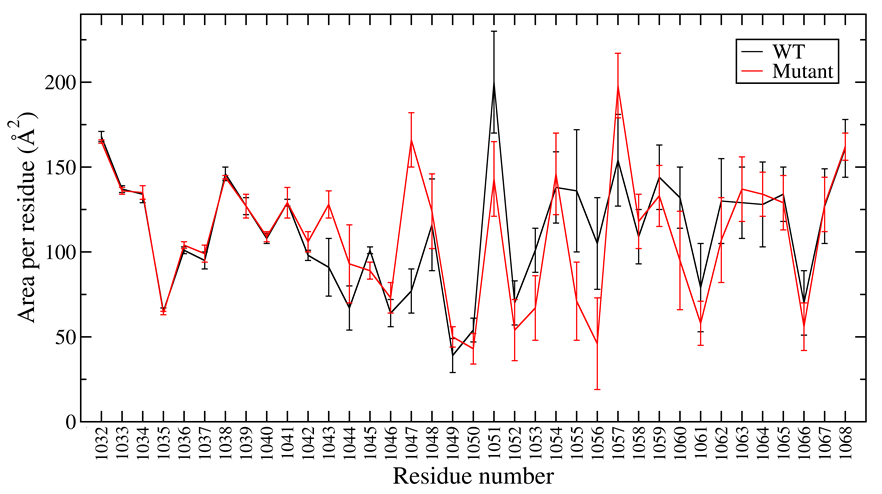

Supplement: Figure S9 — Average solvent accessible area and standard deviation of the C-terminal residues 1032–1068 from five independent unbiased MD simulations for the WT and H1047R mutant proteins. Residues Met-1043, Trp-1057 as well as the mutated residue 1047 have higher solvent accessible area in the mutant, whereas Met-1055 and Asp-1056 are more solvent accessible in the WT protein. (TIF) [file pcbi.1003895.s009.tif]

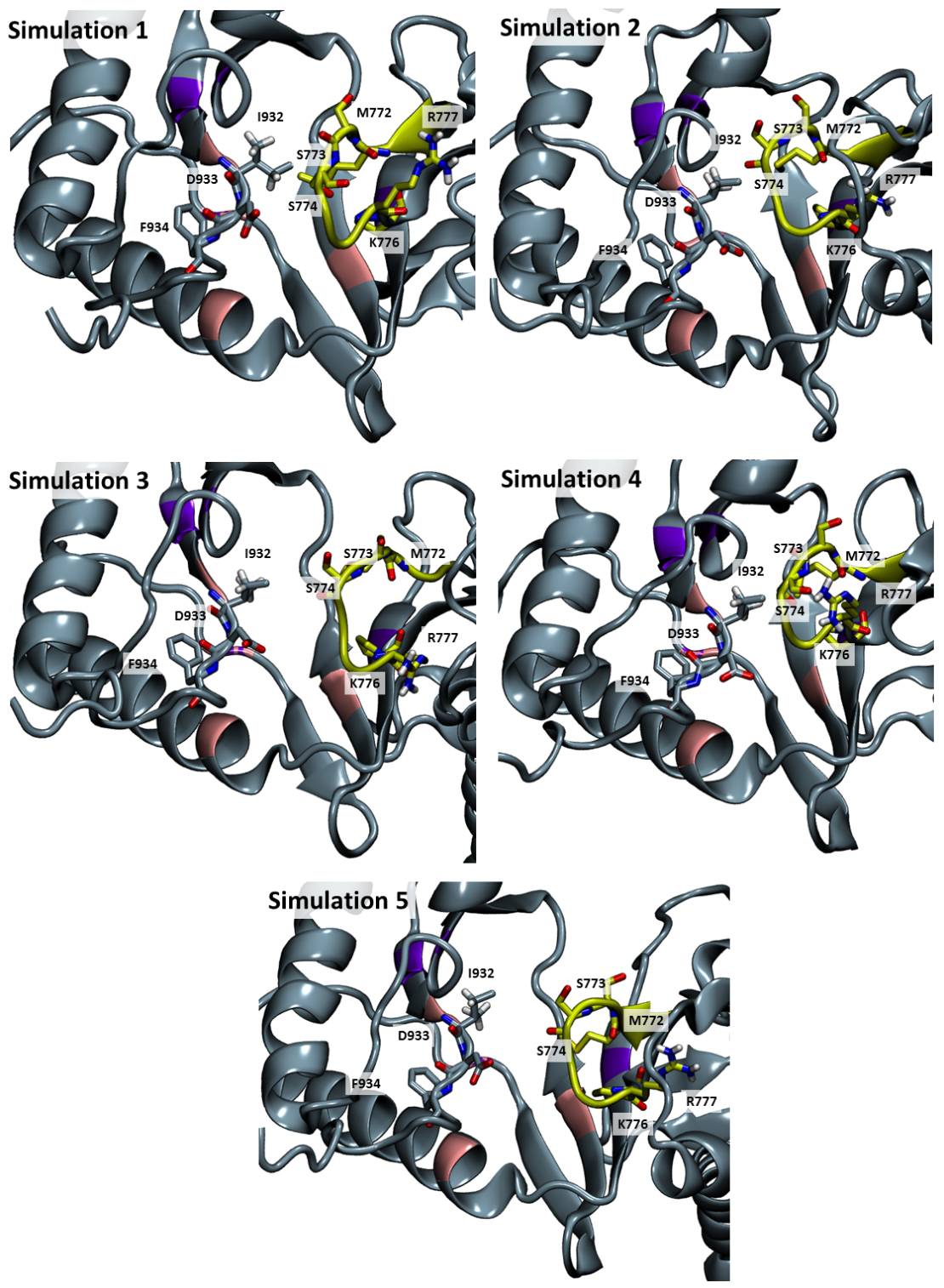

Supplement: Figure S10 — Snapshots close to the active site of the first cluster representatives of the five WT p110α simulations. The P-loop (res. numbers 771–777) is shown in yellow, the affinity pocket (res. numbers 810, 836, 848, 932) in pink, the adenine pocket (res. numbers 800, 836, 922, 930) in orange, and the hinge region (res. numbers 849, 851) is colored in purple. Residues forming hydrogen bonds are shown in licorice representation. (TIF) [file pcbi.1003895.s010.tif]

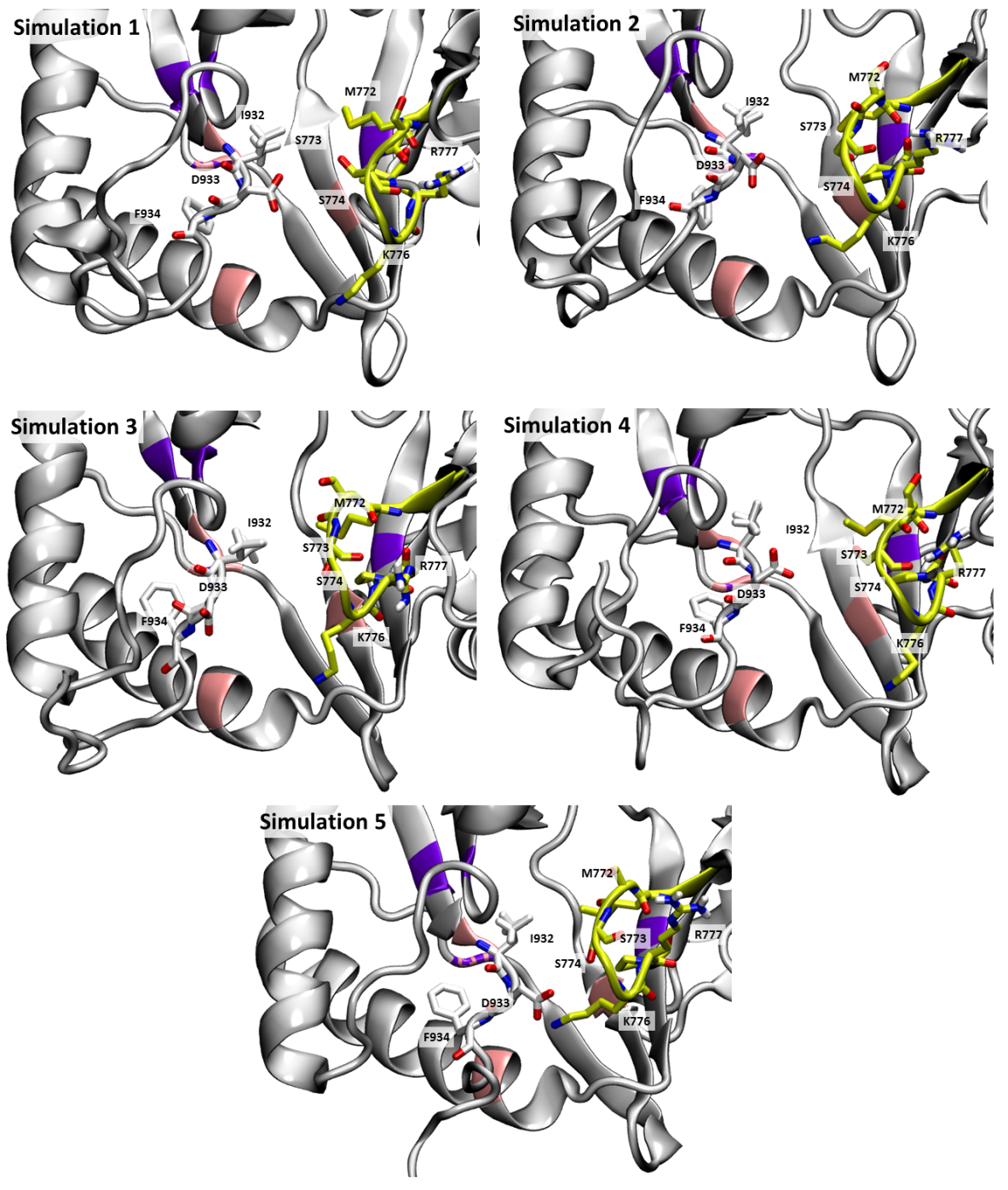

Supplement: Figure S11 — Snapshots close to the active site of the first cluster representatives of the five 1047R p110α simulations. The P-loop (res. numbers 771–777) is shown in yellow, the affinity pocket (res. numbers 810, 836, 848, 932) in pink, the adenine pocket (res. numbers 800, 836, 922, 930) in orange, and the hinge region (res. numbers 849, 851) is colored in purple. Residues forming hydrogen bonds are shown in licorice representation. (TIF) [file pcbi.1003895.s011.tif]

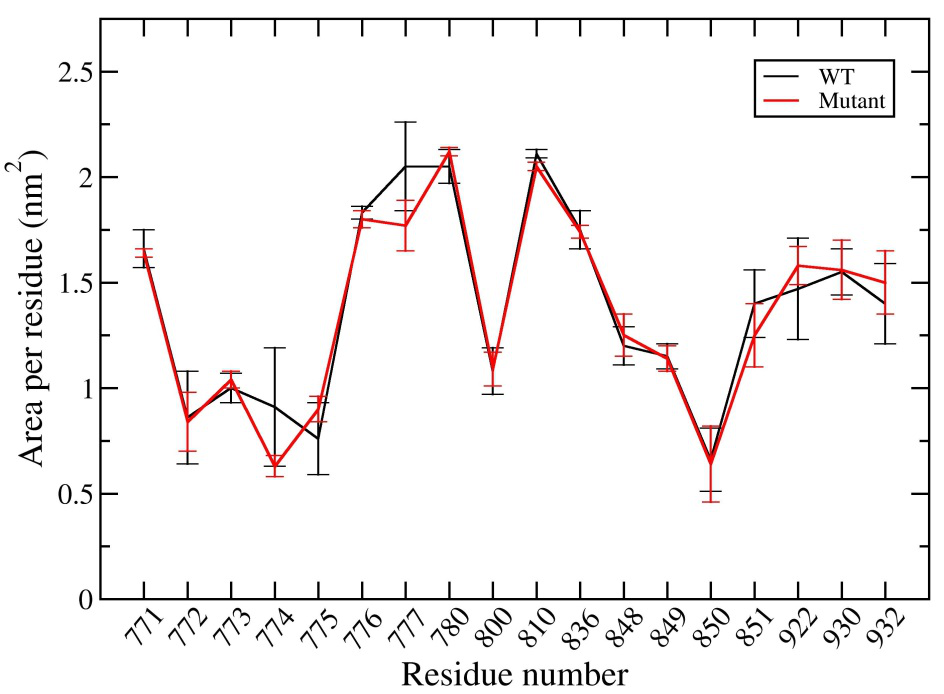

Supplement: Figure S12 — Average solvent accessible area and standard deviation of the active site residues from five independent unbiased MD simulations for the WT and H1047R mutant proteins. Following Williams' notation (Williams et al., 2009), the catalytic site was defined to consist of the P-loop (771–777), the hinge region (849, 851), the adenine pocket (800, 836, 922, 930), the gatekeeper (848), the affinity pocket (810, 836, 848, 932) and the specificity pocket (772, 780). (TIF) [file pcbi.1003895.s012.tif]

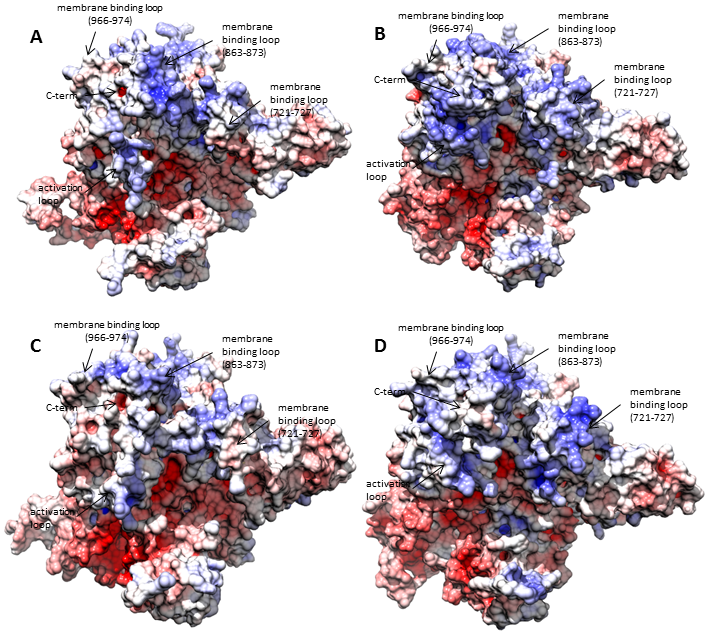

Supplement: Figure S13 — The electrostatic potential on the surface of the average conformation of the WT and H1047R p110α. The images depict membrane interaction areas of the WT (A, C) and H1047R mutant (B, D) cluster 2 and cluster 3 representatives. The surface has been colored with a color scale from −7 eV to 7 eV, with red representing negative charge, white neutral and blue positive. The charge value corresponds to the solvent accessible surface of the protein, namely 1.4 Å far from the surface. These structures were derived from a cluster analysis including all the Cα carbon with a cutoff of 1.7 Å. (TIF) [file pcbi.1003895.s013.tif]

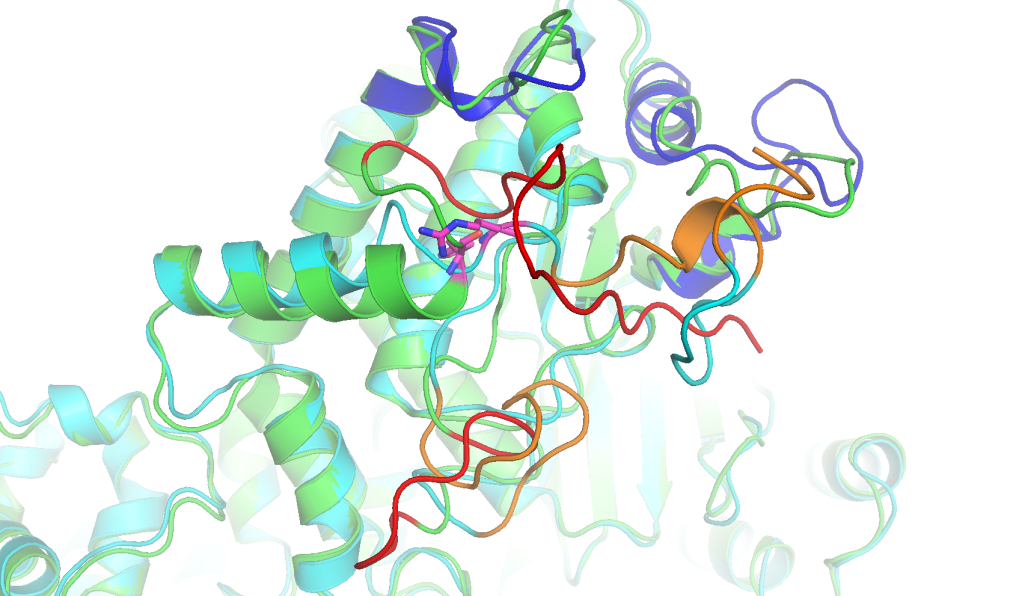

Supplement: Figure S14 — The kinase domain of the same model (cyan) overlaid onto the WT model (green). Residues created through loop modeling are colored orange in the mutant and red in the WT. The initial configuration of the C-termini of the two proteins occupy the same area in space. Residues constructed through homology modeling in the mutant are blue. Arg-1047 and His-1047 are depicted as magenta sticks. (TIF) [file pcbi.1003895.s014.tif]

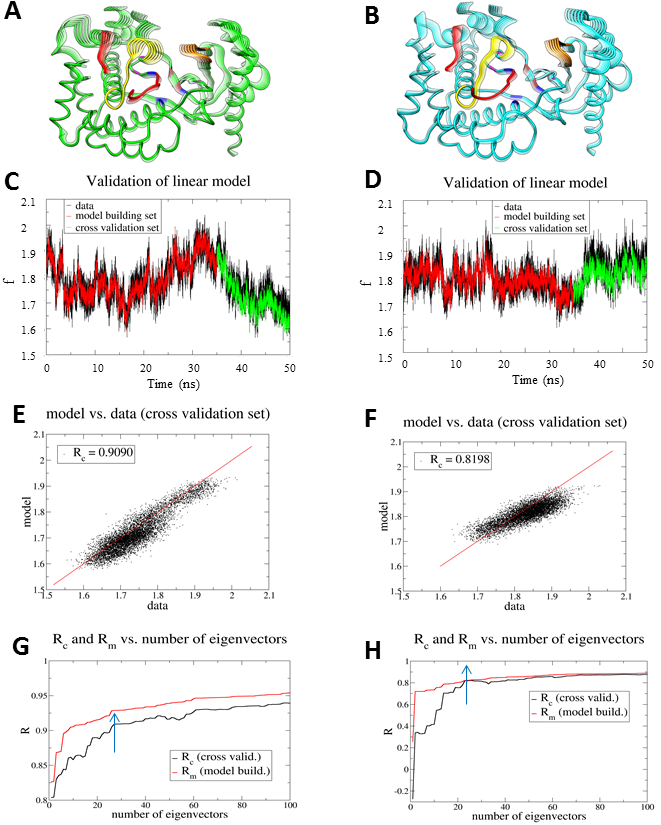

Supplement: Figure S15 — Functional mode analysis of the distance between the Cα carbons of Leu781 and Met922 (dLM). (A/B) the functional mode representing the kinase C- and N-lobe hinge bending motion for the WT and mutant proteins, respectively. Red: activation loop; yellow: catalytic loop; orange: P-loop; brown: hinge; magenta: adenine pocket; blue: affinity pocket (hydrophobic region I); black: specificity pocket. (C/D) the functional quantity f (dLM) versus the simulation time (black curve) for the WT and mutant, respectively. Red and green curves represent the model in the model and cross-validation building set, respectively. (E/F) scatter plots of the data versus the model using the cross-validation sets only. (G/H) Correlations Rm and Rc for dLM as a functions of the number of eigenvectors used during the optimization; blue arrows indicate the number of eigenvectors used for the basis set. (TIF) [file pcbi.1003895.s015.tif]

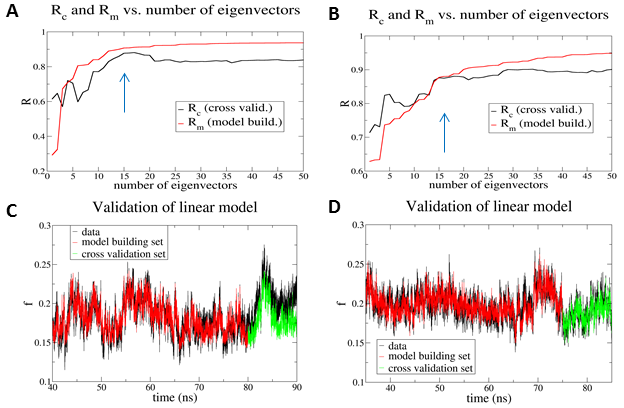

Supplement: Figure S16 — Functional mode analysis of the Cα RMSD of active site residues. (A/B) Correlations Rm and Rc for RMSD as a functions of the number of eigenvectors used during the optimization; blue arrows indicate the number of eigenvectors used for the basis set. (C/D) the functional quantity f (RMSD) in nm versus the simulation time (black curve) for the WT and mutant, respectively. Red and green curves represent the model in the model and cross-validation building set, respectively. (TIF) [file pcbi.1003895.s016.tif]

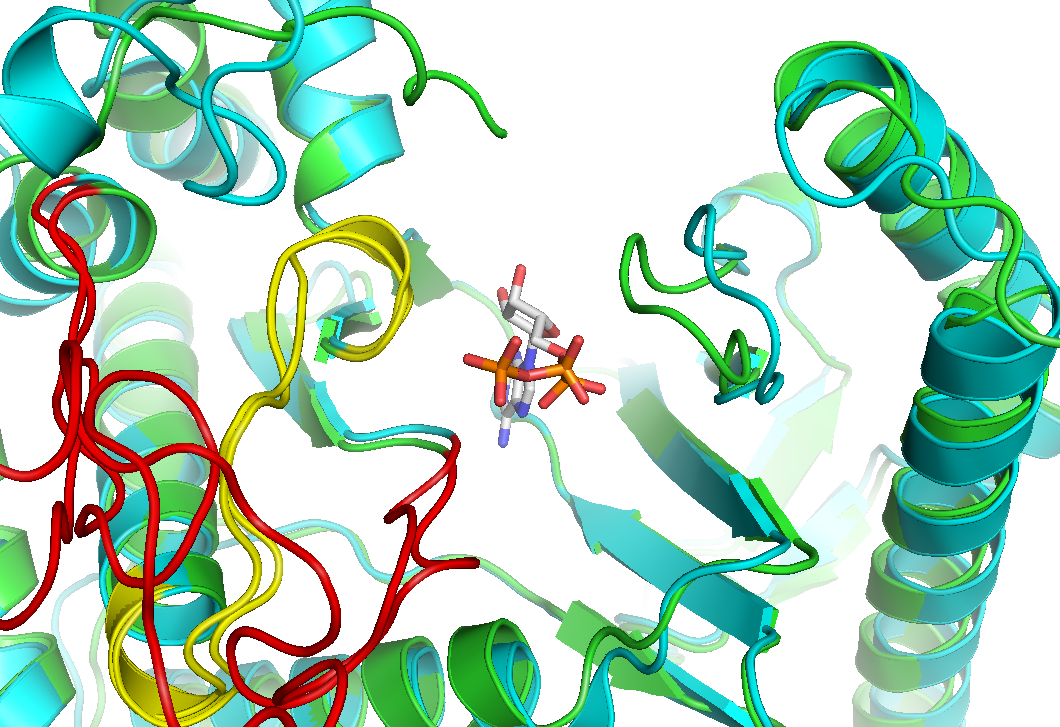

Supplement: Figure S17 — Average conformation of the ATP-binding site of the WT (green) and the H1047R (cyan) p110α. The P-loop of the WT curls inward towards the ATP-binding cavity when compared with the H1047R p110α, so than the active site of the WT is more closed than the mutant. Red: activation loop; yellow: catalytic loop. In the final state, the Cα atoms of the two P-loops are within an average distance of 4.79 Å. The ATP is placed manually to indicate the active site. (TIF) [file pcbi.1003895.s017.tif]

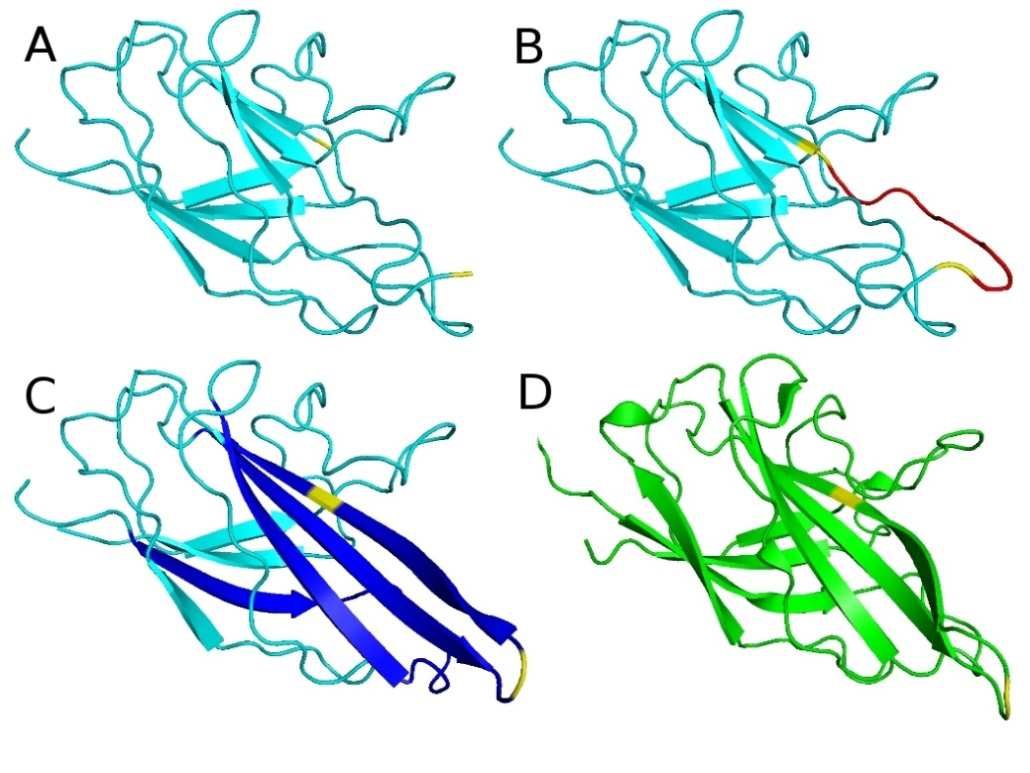

Supplement: Figure S18 — The C2 domain of the human WT p110α. The two ends of the missing loop (positions 414 and 424) are colored yellow. (A) The C2 domain of the WT p110α crystal structure (PDB accession code: 2RD0). (B) The C2 domain of the same structure after loop modeling. The predicted conformation of the loop residues (415–423) is shown in red. (C) The C2 domain of the same structure after homology modeling using the solution NMR structure of the isolated human C2 domain as a template (PDB accession code: 2ENQ). Blue indicates the part that was re-modeled. (D) The solution NMR structure of the isolated human C2 domain (PDB accession code: 2ENQ). (TIF) [file pcbi.1003895.s018.tif]

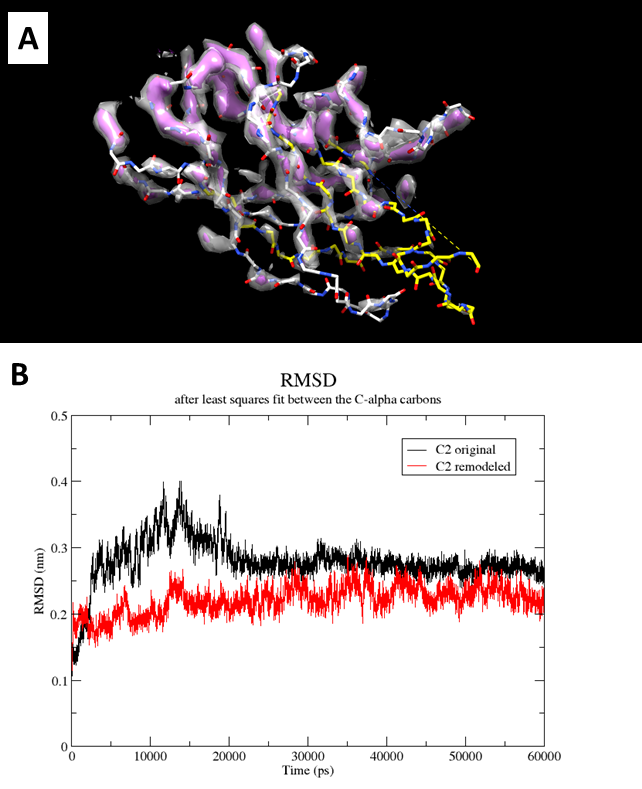

Supplement: Figure S19 — Remodeling of the C2 domain. (A) Electron density around the C2 domain in the WT p110a (PDB code 2RD0). Yellow sticks represent residues that were remodeled. (B) The Cα RMSD with respect to the starting conformation of the original C2 domain (black) versus the remodeled one (red). The remodeled C2 domain converges faster to average RMSD value, meaning that it is closer to equilibrium than the C2 domain in the original crystal structure. (TIF) [file pcbi.1003895.s019.tif]
