# Supplementary material for: Investigating the Structure and Dynamics of the PIK3CA Wild-Type and H1047R Oncogenic Mutant
Source: PLoS Comput Biol. 2014 Oct 23;10(10):e1003895. doi: 10.1371/journal.pcbi.1003895 (PMC4207468; doi:10.1371/journal.pcbi.1003895)
Supplement: Table S1 — List of simulated systems. The analysis of the trajectories was performed in the last 50 ns of each trajectory. (DOCX) [file pcbi.1003895.s020.docx]

**Table S1.** List of simulated systems. The analysis of the trajectories was performed in the last 50 ns of each trajectory.

| **Number** | **System** | **Total simulation**  **length (ns)** |
| --- | --- | --- |
| WT-1 | Human WT | 150 |
| WT-2 | Human WT | 150 |
| WT-3 | Human WT | 150 |
| WT-4 | Human WT | 170 |
| WT-5  MUT-1  MUT-2  MUT-3  MUT-4  MUT-5 | Human WT  Human mutant H1047R  Human mutant H1047R  Human mutant H1047R  Human mutant H1047R  Human mutant H1047R | 150  175  150  180  185  150 |
