# Supplementary material for: Investigating the Structure and Dynamics of the PIK3CA Wild-Type and H1047R Oncogenic Mutant
Source: PLoS Comput Biol. 2014 Oct 23;10(10):e1003895. doi: 10.1371/journal.pcbi.1003895 (PMC4207468; doi:10.1371/journal.pcbi.1003895)
Supplement: Table S2 — Population (in frames) of the first three clusters of the whole p110α subunit (cutoff = 1.7 Å) and the kinase domain (residues 697–1068) (cutoff = 1 Å) for Simulation 1 of the mutant and WT proteins. Although the total population of the first three clusters is comparable, the majority of the frames in the mutant are accumulated in the first cluster, indicating that the H1047R p110α protein visits less conformational states than the WT p110α during production run. The first three clusters include almost the same number of frames and cover the majority of the population (94.1% and 91.6% for the WT versus 92.6% and 90.7% for the mutant). (DOCX) [file pcbi.1003895.s021.docx]

**Table S2.** Population (in frames) of the first three clusters of the whole p110α subunit (cutoff = 1.7Å) and the kinase domain (residues 697-1068) (cutoff = 1 Å) for Simulation 1 of the mutant and WT proteins. Although the total population of the first three clusters is comparable, the majority of the frames in the mutant are accumulated in the first cluster, indicating that the H1047R p110α protein visits less conformational states than the WT p110α during production run. The first three clusters include almost the same number of frames and cover the majority of the population (94.1% and 91.6% for the WT versus 92.6% and 90.7% for the mutant).

|  | **WT p110α** | **H1047R p110α** | **WT kinase domain** | **H1047R kinase domain** |
| --- | --- | --- | --- | --- |
| **Cluster 1** | 16602 | 21921 | 16004 | 16729 |
| **Cluster 2** | 5353 | 2095 | 5281 | 3729 |
| **Cluster 3** | 1580 | 688 | 1608 | 2218 |
| **Percent of the total number of frames** | 94.14% | 98.82% | 91.57% | 90.704 % |
