# Supplementary material for: Investigating the Structure and Dynamics of the PIK3CA Wild-Type and H1047R Oncogenic Mutant
Source: PLoS Comput Biol. 2014 Oct 23;10(10):e1003895. doi: 10.1371/journal.pcbi.1003895 (PMC4207468; doi:10.1371/journal.pcbi.1003895)
Supplement: Table S3 — Important hydrogen bonds and their frequencies in the WT and mutant p110α kinase domain. The hydrogen bonds are sorted by the amino acid in the donor-acceptor pair with the lowest index. Atom names follow the CHARMM force field naming scheme. (DOCX) [file pcbi.1003895.s022.docx]

**Table S3. Important hydrogen bonds and their frequencies in the WT and mutant p110α kinase domain.** The hydrogen bonds are sorted by the amino acid in the donor-acceptor pair with the lowest index. Atom names follow the CHARMM force field naming scheme.

| **Donor** | | | **Acceptor** | | | **WT**  **(%)** | **Mutant**  **(%)** |
| --- | --- | --- | --- | --- | --- | --- | --- |
| ARG770 | P-loop | N | TRP780 | specificity pocket | O | 37.0±7.5 | 73.1±8.8 |
| MET772 |  | N | PRO778 |  | O | 75.0±12.1 | 88.7±1.5 |
| ARG777 |  | N | SER774 | P-loop | O | 11.2±7.9 | 45.2±6.5 |
| TRP780 | specificity pocket | N | ARG770 |  | O | 39.5±7.7 | 86.6±0.9 |
| TRP783 |  | NE1 | LEU748 | activation loop | O | 0.00 | 84.6±1.9 |
| TRP783 |  | N | ASN797 |  | O | 74.4±3.4 | 79.5±5.7 |
| MET858 |  | N | ASN918 | catalytic loop | O | 37.9±3.6 | 44.5±4.1 |
| ARG916 | DRH motif | NH1/NH2 | ASP933 | activation loop | O | 79.2±6.2 | 49.6±14.2 |
| HIS917 | DRH motif | N | ASN920 | catalytic loop | OD1 | 34.1±14.6 | 11.2±9.9 |
| ASN920 | catalytic loop | N | ASD917 | DRH motif | O | 59.3±9.5 | 65.9±2.9 |
| ASN920 |  | ND2 | ILE932 | affinity pocket | O | 42.1±11.5 | 24.1±7.5 |
| ILE932 | affinity pocket | N | ASN920 | catalytic loop | O | 82.8±2.6 | 86.6±1.4 |
| PHE934 | activation loop | N | ASP810 | affinity pocket | OD1/  OD2 | 92.0±1.3 | 42.9±22.8 |
| GLY935 |  | N | ASP933 | activation loop | OD1 | 25.5±4.7 | 3.0±1.8 |
| PHE937 |  | N | PHE934 |  | O | 48.0±3.1 | 0.00 |
| ASP939 |  | N | GLY912 | catalytic loop | O | 45.0±17.0 | 21.6±14.8 |
| ARG949 |  | NE/NH1/  NH2 | ASP915 | DRH motif | OD1/  OD2 | 77.6±6.4 | 0.00 |
| LEU956 |  | N | HIS1047 | kα11 | NE2 | 91.9±1.9 | 0.00 |
| HIS1047 | kα11 | N | MET1043 |  | O | 45.5±7.1 | 0.00 |
| HIS1047 |  | ND1 | MET1043 |  | O | 63.7±4.7 | 8.9±5.5 |
