# Supplementary material for: Investigating the Structure and Dynamics of the PIK3CA Wild-Type and H1047R Oncogenic Mutant
Source: PLoS Comput Biol. 2014 Oct 23;10(10):e1003895. doi: 10.1371/journal.pcbi.1003895 (PMC4207468; doi:10.1371/journal.pcbi.1003895)
Supplement: Table S4 — Average distance and standard deviation in Å from five independent unbiased MD simulations. (DOCX) [file pcbi.1003895.s023.docx]

**Table S4.** Average distance and standard deviation in Å from five independent unbiased MD simulations.

|  | **W-I distance (Å)** | **I-F distance (Å)** |
| --- | --- | --- |
| **WT** | 7.45±1.24 | 6.25±1.38 |
| **H1047R mutant** | 6.82±1.45 | 5.96±0.58 |
