# Supplementary material for: Investigating the Structure and Dynamics of the PIK3CA Wild-Type and H1047R Oncogenic Mutant
Source: PLoS Comput Biol. 2014 Oct 23;10(10):e1003895. doi: 10.1371/journal.pcbi.1003895 (PMC4207468; doi:10.1371/journal.pcbi.1003895)
Supplement: Table S5 — Average area and standard deviation of the C-terminal and catalytic site residues from five independent unbiased MD simulations. (DOCX) [file pcbi.1003895.s024.docx]

**Table S5.** Average area and standard deviation of the C-terminal and catalytic site residues from five independent unbiased MD simulations.

|  | **Average area and standard error (A^2^)** | | |
| --- | --- | --- | --- |
|  | kα12 (res. 1032-1047) | res. 1048-1068 | ATP pocket |
| **WT** | 1862±15 | 2603±42 | 1618±73 |
| **H1047R mutant** | 1957±29 | 2286±42 | 1664±42 |
