# Supplementary material for: Investigating the Structure and Dynamics of the PIK3CA Wild-Type and H1047R Oncogenic Mutant
Source: PLoS Comput Biol. 2014 Oct 23;10(10):e1003895. doi: 10.1371/journal.pcbi.1003895 (PMC4207468; doi:10.1371/journal.pcbi.1003895)
Supplement: Table S6 — Average RMSF values and standard errors (Å) of the functionally important loops of the kinase domain in the WT and H1047R p110α. (DOCX) [file pcbi.1003895.s025.docx]

**Table S6.** Average RMSF values and standard errors (Å) of the functionally important loops of the kinase domain in the WT and H1047R p110α.

|  | **WT** | **Mutant** |
| --- | --- | --- |
| **Mem. Bind. loop 2 (863-873)** | 1.99±0.16 | 1.90±0.17 |
| **Mem. Bind. loop 1 (721-727)** | 2.25±0.10 | 2.57±0.22 |
| **whole Activation loop (933-958)** | 1.26±0.12 | 1.66±0.16 |
| **C-terminus (1048-1068)** | 3.40±0.9 | 3.42±0.36 |
| **Activation loop (933-940)** | 0.77±0.04 | 1.17±0.04 |
| **Activation loop (953-958)** | 1.00±0.05 | 1.4±0.16 |
| **Catalytic loop (909-920)** | 0.81±0.04 | 1.04±0.05 |
| **P-loop (771-777)** | 1.22±0.16 | 1.38±0.06 |
