# Supplementary material for: Investigating the Structure and Dynamics of the PIK3CA Wild-Type and H1047R Oncogenic Mutant
Source: PLoS Comput Biol. 2014 Oct 23;10(10):e1003895. doi: 10.1371/journal.pcbi.1003895 (PMC4207468; doi:10.1371/journal.pcbi.1003895)
Supplement: Table S7 — Overlap of the covariance matrices between the independent runs for both WT and mutant calculated by means of the Root Mean Square Inner Product (RMSIP). (DOCX) [file pcbi.1003895.s026.docx]

**Table S7.** Overlap of the covariance matrices between the independent runs for both WT and mutant calculated by means of the Root Mean Square Inner Product (RMSIP).

| **Run** | **WT1** | **WT2** | **WT3** | **WT4** | **WT5** | **Mut1** | **Mut2** | **Mut3** | **Mut4** | **Mut5** |
| --- | --- | --- | --- | --- | --- | --- | --- | --- | --- | --- |
| **WT1** | **-** | **0.376** | **0.346** | **0.390** | **0.361** | **0.216** | **0.210** | **0.211** | **0.226** | **0.219** |
| **WT2** |  | **-** | **0.401** | **0.415** | **0.372** | **0.232** | **0.237** | **0.233** | **0.242** | **0.238** |
| **WT3** |  |  | **-** | **0.413** | **0.373** | **0.233** | **0.235** | **0.232** | **0.238** | **0.247** |
| **WT4** |  |  |  | **-** | **0.389** | **0.237** | **0.236** | **0.239** | **0.248** | **0.253** |
| **WT5** |  |  |  |  | **-** | **0.222** | **0.217** | **0.221** | **0.230** | **0.223** |
| **Mut1** |  |  |  |  |  | **-** | **0.420** | **0.404** | **0.418** | **0.415** |
| **Mut2** |  |  |  |  |  |  | **-** | **0.427** | **0.419** | **0.426** |
| **Mut3** |  |  |  |  |  |  |  | **-** | **0.423** | **0.440** |
| **Mut4** |  |  |  |  |  |  |  |  | **-** | **0.440** |
| **Mut5** |  |  |  |  |  |  |  |  |  | - |
