# Supplementary material for: Investigating the Structure and Dynamics of the PIK3CA Wild-Type and H1047R Oncogenic Mutant
Source: PLoS Comput Biol. 2014 Oct 23;10(10):e1003895. doi: 10.1371/journal.pcbi.1003895 (PMC4207468; doi:10.1371/journal.pcbi.1003895)
Supplement: Table S8 — Hydrogen bond frequencies within the C-terminal tail (res. numbers 1048–1068) and the activation loop (res. numbers 933–958) of the WT p110α protein. The hydrogen bonds between the two domains are shown in bold. (DOCX) [file pcbi.1003895.s027.docx]

**Table S8.** Hydrogen bond frequencies within the C-terminal tail (res. numbers 1048-1068) and the activation loop (res. numbers 933-958) of the WT p110α protein. The hydrogen bonds between the two domains are shown in bold.

|  | **Acceptor** | **Donor** | **Frequency (%)** |
| --- | --- | --- | --- |
| **Sim1** | ARG949-Main | GLU950-Side | 90.14% |
|  | **THR1053-Side** | **LEU956-Main** | **89.62%** |
|  | GLY946-Main | GLU950-Side | 77.09% |
|  | LYS942-Side | GLU950-Side | 67.88% |
|  | HSD1060-Main | TRP1057-Main | 57.05% |
|  | TYR947-Main | GLU950-Side | 51.12% |
|  | HSD936-Side | PHE937-Main | 49.72% |
|  | PHE937-Main | PHE934-Main | 46.25% |
|  | LYS941-Main | ASP939-Side | 38.62% |
|  | LYS1063-Main | THR1061-Side | 33.73% |
|  | HSD940-Main | ASP939-Side | 27.52% |
|  | LYS1063-Side | ASP1056-Side | 25.95% |
|  | LYS948-Main | GLU950-Side | 23.45% |
|  | **LYS1054-Side** | **ARG951-Main** | **23.43%** |
|  | **GLY935-Main** | **ASP933-Side** | **22.22%** |
|  | LYS941-Side | HSD936-Main | 21.54% |
|  | LYS941-Side | LEU938-Main | 19.88% |
|  | TRP1051-Main | HSD1048-Main | 18.06% |
|  | THR1061-Main | TRP1057-Main | 15.45% |
|  | HSD1048-Side | THR1052-Main | 14.23% |
|  | **ARG949-Side** | **HSD1060-Side** | **12.85%** |
|  | **GLN958-Main** | **LYS1054-Main** | **10.84%** |
|  | THR1061-Side | TRP1057-Main | 10.44% |
|  | THR1061-Side | ASP1056-Side | 10.22% |
| **Sim2** | ARG949-Main | GLU950-Side | 95.96% |
|  | TYR947-Main | GLU950-Side | 56.84% |
|  | LYS948-Main | GLU950-Side | 56.36% |
|  | LYS1054-Main | THR1052-Side | 57.76% |
|  | PHE937-Main | PHE934-Main | 46.30% |
|  | HSD936-Side | PHE937-Main | 46.20% |
|  | **LYS948-Side** | **ASP1056-Side** | **43.74%** |
|  | LYS942-Side | GLU950-Side | 41.84% |
|  | TRP1051-Main | HSD1048-Main | 39.24% |
|  | LYS941-Main | ASP939-Side | 33.97% |
|  | HSD1048-Side | THR1052-Main | 30.29% |
|  | LYS941-Side | HSD936-Main | 22.91% |
|  | LYS941-Side | LEU938-Main | 19.81% |
|  | **GLY935-Main** | **ASP933-Side** | **17.27%** |
|  | HSD940-Main | ASP939-Side | 16.75% |
|  | LYS943-Side | ASP939-Side | 16.71% |
|  | LYS942-Side | ASN1068-Side | 16.69% |
|  | **TYR947-Side** | **THR1061-Main** | **17.25%** |
|  | **ASP1056-Main** | **LYS948-Main** | **14.71%** |
|  | HSD1060-Side | ILE1062-Main | 12.77% |
|  | THR1052-Side | TRP1057-Side | 10.92% |
| **Sim3** | ARG949-Main | GLU950-Side | 97.54% |
|  | HSD940-Main | ASP939-Side | 91.62% |
|  | LYS941-Main | ASP939-Side | 88.84% |
|  | ARG949-Side | ASP939-Main | 78.39% |
|  | HSD940-Side | GLU950-Side | 77.05% |
|  | GLY946-Main | GLU950-Side | 73.11% |
|  | LYS1054-Main | THR1052-Side | 68.51% |
|  | ARG951-Side | TYR947-Side | 49.24% |
|  | PHE937-Main | PHE934-Main | 47.16% |
|  | TYR947-Main | GLU950-Side | 41.60% |
|  | **GLY935-Main** | **ASP933-Side** | **33.17%** |
|  | LYS948-Main | GLU950-Side | 30.63% |
|  | VAL952-Main | GLU950-Main | 25.99% |
|  | ARG949-Side | LEU938-Main | 24.31% |
|  | **THR1053-Side** | **GLN958-Side** | **18.27%** |
|  | LYS942-Side | HSD940-Main | 16.27% |
|  | **GLN958-Side** | **THR1053-Side** | **12.93%** |
|  | **GLN958-Main** | **THR1053-Main** | **10.00%** |
| **Sim4** | **ARG949-Side** | **ASN1068-Side** | **115.85%** |
|  | ARG949-Main | GLU950-Side | 85.31% |
|  | LYS941-Main | ASP939-Side | 74.33% |
|  | **LYS1054-Main** | **LEU956-Main** | **73.69%** |
|  | GLY946-Main | GLU950-Side | 69.33% |
|  | LYS1063-Main | THR1061-Side | 63.81% |
|  | HSD1060-Main | TRP1057-Main | 62.12% |
|  | **GLN958-Main** | **LYS1054-Main** | **57.96%** |
|  | LYS1063-Side | ASP1056-Side | 53.54% |
|  | LYS942-Side | GLU950-Side | 52.96% |
|  | THR1052-Side | GLY1049-Main | 51.62% |
|  | HSD936-Side | PHE937-Main | 51.38% |
|  | VAL952-Main | ARG949-Main | 51.36% |
|  | HSD940-Main | ASP939-Side | 49.18% |
|  | THR1061-Side | ASP1056-Side | 48.82% |
|  | **THR1053-Side** | **GLN958-Side** | **48.56%** |
|  | THR1052-Main | GLY1049-Main | 42.58% |
|  | TYR947-Main | GLU950-Side | 42.36% |
|  | ASN1068-Side | ASP1056-Main | 41.20% |
|  | PHE937-Main | PHE934-Main | 40.94% |
|  | LYS948-Main | GLU950-Side | 36.45% |
|  | **LYS948-Side** | **ASN1068-Side** | **26.69%** |
|  | THR1061-Main | TRP1057-Main | 23.37% |
|  | LYS1054-Side | LEU1067-Main | 15.91% |
|  | **GLY935-Main** | **ASP933-Side** | **15.15%** |
|  | LYS1054-Side | ASP1056-Side | 14.21% |
|  | LYS941-Side | LEU938-Main | 13.45% |
|  | ALA1066-Main | LYS1063-Main | 11.28% |
|  | GLN1064-Side | HSD1065-Side | 11.00% |
| **Sim5** | **ARG949-Side** | **ASP1056-Side** | **143.92%** |
|  | ARG951-Side | ASP939-Side | 129.07% |
|  | **THR1053-Side** | **LEU956-Main** | **88.26%** |
|  | LYS941-Main | ASP939-Side | 59.70% |
|  | PHE937-Main | PHE934-Main | 59.46% |
|  | GLY946-Main | GLU950-Side | 54.18% |
|  | HSD1065-Main | THR1061-Main | 54.04% |
|  | GLN1064-Main | HSD1060-Main | 50.76% |
|  | LYS942-Side | GLU950-Side | 49.90% |
|  | **GLY935-Main** | **ASP933-Side** | **39.68%** |
|  | LEU1067-Main | LYS1063-Main | 32.11% |
|  | ARG949-Main | GLU950-Side | 30.85% |
|  | ALA1066-Main | ILE1062-Main | 29.01% |
|  | HSD940-Side | ASP939-Side | 27.91% |
|  | ARG951-Side | HSD940-Side | 22.67% |
|  | LYS948-Main | GLU950-Side | 22.33% |
|  | ASN1068-Side | GLN1064-Main | 19.21% |
|  | GLU950-Main | GLY946-Main | 18.65% |
|  | ASN1068-Main | HSD1065-Main | 18.17% |
|  | GLN1064-Side | PHE1059-Main | 18.15% |
|  | HSD1060-Side | ILE1058-Main | 16.05% |
|  | GLN1064-Main | THR1061-Main | 15.97% |
|  | LEU1067-Main | GLN1064-Main | 14.77% |
|  | LYS1063-Main | HSD1060-Main | 14.67% |
|  | ARG949-Main | GLY946-Main | 11.64% |
|  | ASN1068-Main | GLN1064-Main | 10.66% |
|  | HSD940-Main | ASP939-Side | 10.06% |
