# Supplementary material for: Investigating the Structure and Dynamics of the PIK3CA Wild-Type and H1047R Oncogenic Mutant
Source: PLoS Comput Biol. 2014 Oct 23;10(10):e1003895. doi: 10.1371/journal.pcbi.1003895 (PMC4207468; doi:10.1371/journal.pcbi.1003895)
Supplement: Table S9 — Hydrogen bond frequencies within the C-terminal tail (res. numbers 1048–1068) and the activation loop (res. numbers 933–958) of the H1047R mutant p110α protein. The hydrogen bonds between the two domains are shown in bold. (DOCX) [file pcbi.1003895.s028.docx]

**Table S9.** Hydrogen bond frequencies within the C-terminal tail (res. numbers 1048-1068) and the activation loop (res. numbers 933-958) of the H1047R mutant p110α protein. The hydrogen bonds between the two domains are shown in bold.

|  | **Acceptor** | **Donor** | **Frequency (%)** |
| --- | --- | --- | --- |
| **Sim1** | **ARG949-Side** | **ASP1045-Side** | **178.71** |
|  | PHE1059-Main | ASP1056-Side | 86.49 |
|  | LYS941-Side | ASP939-Main | 83.73 |
|  | ARG951-Side | GLU950-Side | 76.37 |
|  | LYS943-Main | HSD940-Side | 62.24 |
|  | HSD1060-Side | ASP1056-Main | 61.50 |
|  | ASP1056-Main | THR1052-Main | 60.56 |
|  | TRP1051-Main | HSD1048-Main | 50.16 |
|  | ARG949-Main | GLY946-Main | 48.94 |
|  | LYS1054-Main | GLY1050-Main | 42.80 |
|  | MET1055-Main | TRP1051-Main | 39.24 |
|  | ILE1058-Main | ASP1056-Side | 37.39 |
|  | LYS1063-Side | ASP1056-Side | 32.67 |
|  | LYS943-Side | ASP939-Side | 27.03 |
|  | LYS943-Side | HSD940-Main | 26.61 |
|  | **ARG951-Side** | **ASP939-Side** | **21.87** |
|  | HSD1060-Main | ASP1056-Side | 20.15 |
|  | **THR1061-Side** | **PHE945-Main** | **16.37** |
|  | **LYS942-Side** | **HSD1065-Side** | **14.89** |
|  | **GLN958-Main** | **HSD1060-Side** | **13.55** |
|  | THR1052-Main | HSD1048-Main | 13.21 |
|  | LYS944-Side | LYS942-Main | 11.48 |
|  | THR1061-Main | ASP1056-Side | 10.06 |
| **Sim2** | **ARG951-Side** | **ASP939-Side** | **184.55** |
|  | **ARG949-Side** | **ASP1045-Side** | **148.58** |
|  | LYS944-Side | HSD940-Side | 69.23 |
|  | LYS1054-Main | GLY1050-Main | 68.49 |
|  | LYS941-Main | ASP939-Side | 65.59 |
|  | MET1055-Main | TRP1051-Main | 61.28 |
|  | ARG951-Side | GLU950-Side | 59.94 |
|  | HSD1048-Side | GLY1049-Main | 49.04 |
|  | **ALA1066-Main** | **LYS944-Main** | **45.68** |
|  | LYS1063-Main | THR1061-Side | 41.70 |
|  | HSD940-Main | ASP939-Side | 40.80 |
|  | PHE1059-Main | ASP1056-Main | 38.20 |
|  | HSD1065-Main | THR1061-Side | 35.51 |
|  | LYS943-Side | GLU950-Side | 31.81 |
|  | THR1053-Side | HSD1048-Main | 21.49 |
|  | HSD1065-Side | LEU1067-Main | 19.65 |
|  | **ARG949-Side** | **HSD1048-Side** | **19.55** |
|  | **LYS944-Side** | **GLN1064-Side** | **15.71** |
|  | LYS941-Side | ASP939-Main | 15.37 |
|  | ARG949-Side | ARG951-Main | 14.91 |
|  | THR1061-Side | ASN1068-Side | 12.77 |
|  | **TYR947-Main** | **ASN1068-Side** | **11.62** |
|  | LYS941-Side | ASP939-Side | 10.14 |
| **Sim3** | ARG949-Side | GLU950-Side | 119.83 |
|  | PHE1059-Main | ASP1056-Side | 88.28 |
|  | ASP1056-Main | THR1052-Main | 75.87 |
|  | ARG949-Main | GLY946-Main | 75.09 |
|  | GLY946-Main | LYS943-Main | 73.65 |
|  | THR1053-Side | GLY1049-Main | 70.97 |
|  | ILE1058-Main | ASP1056-Side | 63.41 |
|  | THR1053-Main | GLY1049-Main | 62.44 |
|  | HSD1060-Side | ASP1056-Main | 48.72 |
|  | MET1055-Main | TRP1051-Main | 46.48 |
|  | TYR947-Main | LYS944-Main | 37.66 |
|  | LYS941-Side | ASP939-Side | 36.97 |
|  | LYS1054-Main | GLY1050-Main | 35.97 |
|  | LEU1067-Main | LYS1063-Main | 33.69 |
|  | HSD1060-Main | ASP1056-Side | 25.55 |
|  | LYS1063-Side | ASP1056-Side | 24.07 |
|  | THR1052-Main | HSD1048-Main | 23.75 |
|  | LYS942-Side | HSD940-Side | 19.89 |
|  | ALA1066-Main | LYS1063-Main | 18.25 |
|  | HSD1065-Main | ILE1062-Main | 16.75 |
|  | ASN1068-Main | GLN1064-Main | 14.51 |
|  | ASN1068-Side | GLN1064-Main | 11.98 |
|  | ALA1066-Main | ILE1062-Main | 11.82 |
| **Sim4** | PHE1059-Main | ASP1056-Side | 83.85 |
|  | LYS941-Side | GLU950-Side | 67.39 |
|  | ASP1056-Main | THR1052-Main | 66.69 |
|  | MET1055-Main | TRP1051-Main | 63.71 |
|  | HSD1060-Side | ASP1056-Main | 63.09 |
|  | ARG949-Side | ASP939-Side | 62.20 |
|  | LYS941-Side | HSD940-Side | 60.20 |
|  | HSD940-Main | PHE937-Main | 51.42 |
|  | ILE1058-Main | ASP1056-Side | 51.18 |
|  | LYS1054-Main | GLY1050-Main | 40.42 |
|  | LYS942-Main | TYR947-Main | 39.40 |
|  | HSD1060-Main | ASP1056-Side | 34.13 |
|  | THR1061-Side | LYS1063-Main | 34.01 |
|  | LYS941-Main | ARG949-Main | 23.67 |
|  | TYR947-Main | LYS942-Main | 20.53 |
|  | **TRP1051-Side** | **LEU956-Main** | **18.37** |
|  | GLY946-Main | LYS943-Main | 17.53 |
|  | PHE945-Main | LYS942-Main | 16.41 |
|  | THR1061-Main | ASP1056-Side | 14.11 |
|  | LYS1063-Main | THR1061-Side | 12.18 |
|  | ARG951-Side | GLU950-Side | 10.62 |
| **Sim5** | **ARG949-Side** | **ASP1045-Side** | **160.34** |
|  | **ARG951-Side** | **ASP939-Side** | **121.19** |
|  | ASP1056-Main | THR1052-Main | 67.87 |
|  | PHE1059-Main | ASP1056-Side | 64.83 |
|  | ARG949-Side | LYS948-Main | 52.98 |
|  | MET1055-Main | TRP1051-Main | 50.50 |
|  | LYS942-Side | ASP939-Side | 48.50 |
|  | TYR947-Main | LYS943-Main | 44.86 |
|  | HSD1060-Side | ASP1056-Main | 41.56 |
|  | LYS1063-Side | ASP1056-Side | 41.30 |
|  | LYS948-Main | LYS944-Main | 32.73 |
|  | ILE1058-Main | ASP1056-Side | 30.09 |
|  | LYS1054-Main | GLY1050-Main | 29.63 |
|  | LYS944-Side | GLU950-Main | 29.35 |
|  | **THR1052-Side** | **PHE945-Main** | **24.83** |
|  | ALA1066-Main | LYS1063-Main | 24.57 |
|  | THR1061-Main | ASP1056-Side | 23.49 |
|  | THR1053-Side | GLY1049-Main | 18.29 |
|  | LYS941-Side | HSD940-Side | 17.15 |
|  | GLN1064-Side | HSD1065-Side | 13.79 |
|  | LYS1054-Side | ASN1068-Side | 13.15 |
