# Supplementary material for: Investigating the Structure and Dynamics of the PIK3CA Wild-Type and H1047R Oncogenic Mutant
Source: PLoS Comput Biol. 2014 Oct 23;10(10):e1003895. doi: 10.1371/journal.pcbi.1003895 (PMC4207468; doi:10.1371/journal.pcbi.1003895)
Supplement: Table S10 — Salt-bridge frequencies in the WT and mutant p110α kinase domain. The salt-bridges are sorted by the amino acid in the donor-acceptor pair with the lowest index. (DOCX) [file pcbi.1003895.s029.docx]

**Table S10. Salt-bridge frequencies in the WT and mutant p110α kinase domain.** The salt-bridges are sorted by the amino acid in the donor-acceptor pair with the lowest index.

| **Donor-Acceptor** | **WT (%)** | **Mutant (%)** | **Location** | |
| --- | --- | --- | --- | --- |
| GLU1037-LYS1041 | 16.8±2.2 | 13.6±2.6 | kα11 | kα11 |
| GLU1034-LYS1030 | 43.6±6.5 | 55.0±6.6 | kα11 | kα11 |
| GLU1032-ARG992 | 0.00 | 33.9±9.4 | kα11 | kα8 |
| ASP1029-ARG1023 | 82.9±4.9 | 98.0±0.7 | - | kα10 |
| GLU1012-LYS941 | 52.9±15.6 | 0.00 | - | activation loop |
| GLU982-ARG975 | 57.0±21.3 | 35.3±17.2 | kα8 | kα8 |
| GLU982-LYS986 | 8.4±2.7 | 13.1±2.8 | kα8 | kα8 |
| GLU978-ARG975 | 0.00 | 26.8±13.4 | kα8 | - |
| GLU976-ARG979 | 64.4±12.3 | 25.2±11.0 | kα8 | kα8 |
| GLU976-LYS966 |  | 19.0±4.6 | kα7 | kα7 |
| GLU970-LYS973 | 50.1±12.9 | 36.0±11.4 | - | - |
| GLU950-LYS942 | 51.0±12.8 | 0.00 | activation loop | activation loop |
| ASP933-LYS802 | 34.2±20.8 | 35.8±17.0 | DFG motif | - |
| ASP926-LYS924 | 96.1±1.1 | 85.8±10.0 | - | - |
| ASP925-LYS924 | 87.9±5.9 | 84.6±9.4 | - | - |
| ASP925-LYS884 | 48.1±16.0 | 28.6±11.6 | - | - |
| ASP895-ARG899 | 64.4±7.4 | 71.8±11.7 | - | - |
| ASP891-LYS966 | 0.0 | 79.0±4.2 | - | kα7 |
| GLU888-LYS886 | 0.0 | 16.2±3.6 | - | - |
| ASP830-ARG832 | 61.3±20.2 | 69.2±20.1 | - | - |
| ASP806-ARG808 | 99.8±0.2 | 99.2±0.6 | - | - |
| ASP805-LYS802 | 83.6±7.9 | 51.7±19.2 | - | - |
| ASP805-LYS776 | 79.3±8.6 | 83.3±9.0 | - | P-loop |
| GLU798-ARG770 | 75.3±14.1 | 38.9±17.2 | - | close to P-loop |
| GLU791-ARG765 | 58.8±8.5 | 0.0 | - | - |
| GLU784-ARG765 | 87.1±8.0 | 35.5±14.2 | - | - |
| GLU737-ARG740 | 90.5±5.8 | 75.6±9.8 | - | - |
