# Supplementary material for: Investigating the Structure and Dynamics of the PIK3CA Wild-Type and H1047R Oncogenic Mutant
Source: PLoS Comput Biol. 2014 Oct 23;10(10):e1003895. doi: 10.1371/journal.pcbi.1003895 (PMC4207468; doi:10.1371/journal.pcbi.1003895)
Supplement: Text S1 — The supporting information text provides details on the model construction and refinement. (DOCX) [file pcbi.1003895.s030.docx]

**Supporting Information Text S1**

Investigating the Structure and Dynamics of the *PIK3CA* Wild-Type and H1047R Oncogenic Mutant

Paraskevi Gkeka^1#^, Thomas Evangelidis^1#^, Maria Pavlaki^2^, Vasiliki Lazani^3^, Savvas Christoforidis^3,4^, Bogos Agianian^2^, Zoe Cournia^1,^*

^1^Biomedical Research Foundation, Academy of Athens,,Athens, Greece.

^2^Department of Molecular Biology and Genetics, Democritus University of Thrace, Alexandroupolis, Greece

^3^Department of Biomedical Research, Institute of Molecular Biology and Biotechnology-Foundation for Research and Technology (IMBB-BR/FORTH), Ioannina, Greece

^4^Department of Medicine, University of Ioannina, Ioannina, Greece

# these authors contributed equally to this work

*corresponding author, [zcournia@bioacademy.gr](mailto:zcournia@bioacademy.gr)

Tel: +302106597195, Fax: +302106597545

# A. Methods

A1. Model Construction and Refinement

The amino acid sequence of the WT p110α subunit was retrieved from the UniProt databank (Jain et al., 2009) (UniProt Accession code P42336) in order to construct the full length atomistic model of (a) the WT human p110α (Model 1) and (b) the p110α H1047R mutant (Model 2). The experimentally-determined amino-acids were constrained to their initial configuration, while the missing loops were created either by loop or homology modeling. More specifically, Model 1 was built from PDB structure 2RD0, which lacks residues 1-7, 307-324, 415-423, 506-527, 941-950, and 1051-1068. Two models of the WT p110α were constructed: in Model 1a the missing C2 domain loop residues 415-423 (Figure S1) were created through loop modeling. In Model 1b, residues 335-361 and 401-428 were reconstructed through homology modeling (Figure S18 C), using as a template the solution NMR structure of the human C2 domain with PDB accession code 2ENQ (Figure S18 D), due to the low electron density of the 2RD0 structure at this area (Figure S19 A). The rest of the missing loops of 2RD0 were created through loop modeling (see section A2 below), and share the same coordinates in both Models 1a and 1b. Model 2 was built from the PDB structure 3HIZ, which lacks residues 1-4, 310-320, 418-420, 517-523, 863-872, 941-950, 971, 1049-1055, 1063-1068. C2 domain residues 335-361 and 402-428 (including the missing loop at position 418-420) were rebuilt from PDB accession code 2ENQ through homology modeling, while residues 857-884 (including the missing loop at 863-872) were rebuilt through homology modeling using PDB structure 3HHM as a template due to low electron density in this region (section A3). A large gap between residues 970 and 972 was observed due to the missing residue Cys-971 in PDB structure 2RD0 and therefore the whole region around the missing Cys-971 (residues 963-978) was reconstructed from PDB structure 3HMM through homology modeling. The rest of the missing loops were created through loop modeling. The side-chains of the modeled residues were optimized with SCWRL4 (Krivov et al., 2009) and relaxed using the Molecular Modelling Tool Kit plugin of UCSF Chimera 1.5.2 (Pettersen et al., 2004) using 500 steps of steepest descent energy minimization and an initial step length of 2 Å. During side-chain optimization and energy minimization, the experimentally-determined parts of the structures were constrained to their initial conformation. In the case of H1047R p110α, a broader region of the C2 domain, namely residues 335-386 and 402-453, was selected for smooth energy minimization and side-chain optimization in order to alleviate the steric clashes with their neighboring aminoacids. The modeled regions on the WT and H1047R p110α structure are illustrated in Figure S1.

## A2. Loop Modeling

For the loop modeling of the p110α subunits (WT and H1047R) the following procedure was pursued: Amino-acids within 20 Å from the missing loop ends (loop regions) were isolated from the templates (2RD0, 3HIZ) and saved into new .pdb files (hereafter referred as loop templates) to speed up the calculations and allow for broader conformational sampling. All residues present in the crystal structures were constrained to their initial coordinates. Some loops required up to 4 successive flanking residues to be left flexible in order to allow loop closure. Subsequently, 1000 loop decoys were generated for loops sized 10 residues or less, using MODELLER 9v8 (Sali & Blundell, 1993) and the DOPE-HR (Discrete Optimized Protein Energy in Hight Resolution) loop modeling method (Shen & Sali, 2006). The loop models were refined with conjugate gradient minimization and Molecular Dynamics (MD) simulations with simulated annealing, using the slowest and most accurate protocol of MODELLER (*dopehr_loopmodel.loop.md_level = refine.very_slow*). For loops longer that 10 residues, 3000 loop decoys were generated following the same procedure. The DOPE-HR statistical potential was implemented to estimate a pseudo-energy value (the lowest the better) for each loop decoy, and the one with the lowest raw DOPE-HR score was selected in each case.

## A3. Homology Modeling

For the homology modeling of the C2 domain of both WT and H1047R p110α, as well as residues 857-884 and 963-978 of the H1047R p110α, the following procedure was pursued: Sequence alignments were created manually in Jalview 2.6.1 (Waterhouse et al., 2009) and were passed to MODELLER 9v8 to create 160 homology models from each alignment. Every homology model was obtained by optimization of a molecular probability density function (pdf) using the slowest degree of the variable target function method (VTFM; Braun & Gō, 1985) in Cartesian space (*automodel.library_schedule = autosched.slow*) and setting the maximum number of VTFM iterations to 300 (*automodel.max_var_iterations = 300*). Then, each model was refined with conjugate gradient minimization and MD simulations with simulated annealing using the slowest protocol (*automodel.md_level = refine.very_slow*). The homology models were evaluated using the DOPE-HR statistical potential (Shen & Sali, 2006); the model with the lowest raw score (free energy estimate) was selected. Each full-length p110α subunit was assembled from the respective PDB structure and the selected loop and homology models through homology modeling. The experimentally-determined amino-acids were constrained to their initial conformation.

A4. Molecular Dynamics Simulations

After model construction, each system was subsequently energy-minimized in three consecutive 10,000 conjugate-gradient steps: First, all protein atoms remained fixed using a 500 kcal/(mol^∙^Å^2^) force constant. Then, only backbone atoms were kept fixed with the same force constant, and finally all atoms were left unconstrained. The systems were then gradually heated under constant volume from 0 to 310 K using 1 kcal/(mol^∙^Å^2^) constraints applied on the Cα atoms. The production runs were finally performed without constraints, under constant pressure and temperature (NPT), leading to a total simulation time of ~1µs for the WT with the reconstructed C2 domain, 60 ns for the WT with the original C2 domain and ~1µs for the mutant. A 2 fs time step was used and non-bonded forces were cut off at 12 Å using the CHARMM switched potential between 10-12 Å. Periodic boundary conditions were applied and the Particle Mesh Ewald method (Darden et al., 1993) was used to calculate electrostatic interactions every 4 fs. The pressure was maintained at 1 atm applying the Langevin piston method (Feller et al., 1995), while the temperature was kept at 310 K using Langevin dynamics with a damping coefficient of 5 ps^-1^. Atomic coordinates of the systems were saved every 2 ps.

**A5. Trajectory Analysis**

The trajectories were analyzed with GROMACS tools v4.5.5 [31] to identify predominant cluster conformations, estimate root mean square fluctuation (RMSF) per Cα atom, root mean square deviation (RMSD), hydrogen bond frequencies, solvent accessible surface area (SASA), and to perform principal component analysis (PCA). The electrostatic potential was computed with the APBS algorithm. Functional Mode Analysis (FMA) was performed as discussed in Ref. [35]. Two separate cluster analyses were performed using the gromos algorithm (Daura et al., 1999). The first was applied on the whole protein and the second was focused on the kinase domain (residues 697-1068). The highly flexible loop residues 1-7, 231-240, 291-330, 410-417, 505-530, 863-872, 941-952, 1047-1068 were excluded from the calculations. A cutoff of 1 Å was chosen for the kinase domain and 1.7 Å for the whole protein, as these values produced balanced clusters. The RMSF was computed for the whole protein as well as solely for the kinase domain. Salt bridge frequencies were computed using the “Salt Bridges” plugin of VMD v1.9 [34]. A salt bridge was considered to be formed if the distance between any of the oxygen atoms of acidic residues (GLU, ASP) and the nitrogen atoms of basic residues (ARG, LYS) was within 3.2 Å in at least one frame. The salt bridge frequencies were computed by dividing the number of frames where this distance was lower than 4 Å by the total number of frames. The specific cutoff was chosen based on relevant literature where Potential of Mean Force (PMF) calculations showed that the stabilization of an Arg-Glu salt-bridge, i.e. the minimum of the PMF, occurs at approximately 4 Å (Okur et al., 2008).

In order to identify large-scale correlated motions from random thermal fluctuations in our systems we employed the PCA method. The steps of PCA are: (a) removal of global translational and rotational motions, (b) calculation of the variance-covariance matrix of the interatomic fluctuations, and (c) diagonalization of the matrix (Hayward et al., 2008). PCA was performed on the whole protein as well as on the kinase domain (residues 697-1068). In both cases only the Cα carbon coordinates were used. Loops that were loosely connected to the rest of the protein (residues with high RMSF) were excluded from the calculations in order to extract pure low-frequency motions of the molecule (Hayward et al., 2008). These loops encompass residues 1-7, 231-240, 291-330, 410-417, 505-530, 863-872, 941-952, 1047-1068.

Briefly, following removal of global translational and rotational motions of the protein elements of the variance-covariance matrix of positional fluctuations are calculated from the *3N* cartesian atomic coordinates *x_i_(t), i,j=1,…,3N,* using the following formula:

$C\text{ij}= \left\langle\left( x\text{i}-\left\langle x\text{i} \right\rangle\right)\cdot\left( x\text{j}-\left\langle x\text{j} \right\rangle\right) \right\rangle$,

where *x_1_,…,x_3N_* are the mass-weighted Cartesian coordinates of the atoms and < > is the average over all conformations sampled during the simulation. *C* is a symmetric *3Nx3N* matrix that can be diagonalized by an orthogonal coordinate transformation matrix, *T*:

$C=T\Lambda T^{T}$,

where *Λ* is the diagonal (eigenvalue) matrix and *T* is a matrix with the orthonormal eigenvectors of *C* as its columns. Each eigenvector, *µ_i_*, has a corresponding eigenvalue *λ_i_*, which denotes the mean square coordinate ﬂuctuation in the direction of the eigenvector, *µ_i_*. The trajectory can be projected onto the eigenvectors to give the principal components *p_i_(t), i=1,…,3N* by:

$p\text{i}\left( t \right)=\mu\text{i}\cdot(x\left( t \right)-\left\langle x \right\rangle)$,

where *µ_i_* is the *i^th^* eigenvector of *C* (the *i^th^* column of *T*).

To calculate the electrostatic potential, representative structures from the first three clusters were used. Charges were added with PDB2PQR (Dolinsky et al., 2007) using the “parse” force field, and an input file with the default options was generated for subsequent electrostatic potential calculation with APBS (Baker et al., 2001). FMA was also used to extract collective motions related to WT and/or H1047R protein specific function, following the procedure described in Ref. (Hub & de Groot, 2009) (see also section A5, SI). Binding site predictions were performed with the QSiteFinder web server (Laurie et al., 2005). Images were created with PyMOL v1.4.1 (The PyMOL Molecular Graphics System, Version 1.5.0.4 Schrödinger, LLC) and UCSF Chimera v1.7 (Pettersen et al., 2004), videos with VMD v1.9 and UCSF Chimera v1.7, and plots with the GRaphing, Advanced Computation and Exploration (GRACE) program (Grace Development Core Team, 2011).

The Root Mean Square Inner Product (RMSIP) between the trajectory eigenspaces was calculated using:

$RMSIP= \sqrt{\frac{1}{M}\sum_{i=1}^{M} \sum_{j=1}^{M} (\eta_{i}\cdot\nu_{j})}$ ,

where $\eta_{i}$and $\nu_{j}$ are the *i*th and *j*th eigenvectors of two different subparts, respectively, and *M* is the dimension of the subspaces.

**A6. Functional Mode Analysis**

We consider a simulation trajectory with positions ***x****(t)*∈*R^3N^*, where *N* is the number of atoms and time *t*∈{t_1_,…,t_Nt_} with *N_t_* the times for which the coordinates of the system are known. Let *f(t)* be an arbitrary scalar functional quantity, which can be computed from the protein coordinates (e.g. the active site RMSD). The aim is to find a normalized collective vector **α**∈*R^3N^* of protein atoms such that the motion along **α** is maximally correlated to the change in the functional quantity *f(t)* (Maximally Correlated Motion, MCM). MCM is given by the projection

$$p_{\alpha}=\left[ \boldsymbol{x}\left( t \right)-<\boldsymbol{x}> \right]\cdot\boldsymbol{a}\mathbf{,}$$

where <…> denotes the average over all times *t*.

The correlation between *f* and *p_α_* can be either linear or non-linear. To quantify the linear correlation, the Pearson’s correlation coefficient is used, which is defined by:

$R=\frac{cov\left( f,p\text{α} \right)}{\sigma\text{f}\sigma\text{α}}$,

where *cov(f,p_α_)* is the covariance between *f(t)* and *p_α_(t)*, and *σ_f_* and *σ_α_* are the standard deviations of *f(t)* and *p_α_(t)*, respectively. To quantify non-linear, higher order correlation, the mutual information (MI) between *f* and *p_α_* is used (Cover & Thomas, 1991) given by:

$$I\left( f,p\text{α} \right)=\iint P\left( f^{'},p^{'}\text{α} \right)\log\left( \frac{P\left( f^{'},p^{'}\text{α} \right)}{P\text{1}\left( f^{'} \right)P\text{2}\left( p^{'}\text{α} \right)} \right)df^{'}dp^{'}\text{α},$$

where *P(f´,p´_α_)* denotes the joint probability distribution of *f* and *p_α_*, and *P_1_(f´)* and *P_2_(p´_α_)* denote the marginal probability distributions of *f* and *p_α_*, respectively.

We implemented the FMA technique in order to identify collective motions related to the hinge bending motion that describes the opening and closing of the active site cleft and the C- and N- lobe twisting motions. These collective motions can be described in terms of alteration in a chosen functional quantity, such as the volume of the binding site, the number of hydrogen bonds between two groups, or the distance between two important functional residues. For PI3Kα several functional quantities were attempted, such as the SASA of the active site, distances between functionally relevant residues in the two lobes, volume of the catalytic cleft, the Cα RMSD of the kinase domain as well as each lobe individually. The functional quantity that yielded the highest correlation to the hinge bending motion was found to be the distance (d_LM_) between the Cα carbons of Leu-781 and Met922 of the active site. Residues Leu-781 and Met-922 were selected to quantify the hinge bending motion because they lie on the surface of the opposite sites of the catalytic cleft and their distance is directly related to the opening and closing of the active site.

For d_LM_, the collective vector ***α*** was optimized by maximizing the Pearson’s correlation coefficient (R), yielding linear models for the WT and mutant d_LM_. We used the first 35 ns of the production phase for model building and the rest 15 ns for cross-validation. To avoid over-fitting of the model in the selection of the basis set, the Pearson’s correlation coefficients of the model-building (Rm) and the cross-validation set (Rc) were plotted as a function of the number of eigenvectors used as a basis set (Figures S15A and S15B). We selected the first 27 eigenvectors for the WT and the first 23 for the mutant as both Rm and Rc increase linearly and reach a minimum distance at these points (Figure S15). After these points both Rc and Rm improve in slower rates and the distance between them does not change significantly. These two basis sets yielded a Pearson’s correlation values of 0.91 and 0.82 for the WT and the mutant trajectory, respectively (Figure S15), which denote high correlation between the d_LM_ and the hinge bending motion. The robustness of the two models is demonstrated by the large overlap between the time series of the predicted and real d_LM_ values (Figure S15).

For the description of the twisting motion, the RMSD_active_ was optimized by maximizing the mutual information (MI) coefficient (see section A5, SI for more details). We used the first 40 ns of the production phase for model building the last 10 ns for cross-validation. For the optimization of the non-linear model with the MI we used less than 20 eigenvectors to avoid over-fitting. As shown in Figure S16, the difference between Rm and Rc reaches a minimum when the number of used eigenvectors is 17 in the WT and 13 in the mutant. The two basis sets yielded a Pearson’s correlation value of 0.86 and 0.87 for the WT and the mutant trajectory respectively (Figures 7 and S16), which denote high correlation between the Cα RMSD and the twisting motion of the kinase lobes. The robustness of the two models is demonstrated by the large overlap between the predicted and the real Cα RMSD values (Figure S16).

**B. References**

Baker N, Sept D, Joseph S, Holst M, McCammon J (2001) Electrostatics of nanosystems: Application to microtubules and the ribosome. *Proc Natl Acad Sci USA* 98:10037–10041.

Braun W, Gō N (1985) Calculation of protein conformations by proton-proton distance constraints. A new efficient algorithm. *J Mol Biol* 186:611–626.

Cover TM, Thomas JA (1991) *Elements of Information Theory*, John Wiley & Sons.

Darden T, York D, Pedersen L (1993) Particle mesh Ewald: An N⋅log(N) method for Ewald sums in large systems *J Chem Phys* 98:10089-10093.

Daura X, Gademann K, Jaun B, Seebach D, van Gunsteren WF, Mark AE (1999) Peptide Folding: When Simulation Meets Experiment. *Angewandte Chemie, International Edition* 38, 236-240.

Dolinsky TJ, *et al.* (2007) PDB2PQR: expanding and upgrading automated preparation of biomolecular structures for molecular simulations. *Nucleic Acids Res* 35:W522– W525.

Feller SE, Zhang Y, Pastor RW, Brooks BR (1995) Constant pressure molecular dynamics Simulation: The Langevin piston method. *J Chem Phys* 103: 4613-4621.

Hayward S, de Groot BL (2008) Normal Modes and Essential Dynamics. In Methods in molecular biology; Clifton, N.J., Humana Press, 443:89-106.

Humphrey W, Dalke A, Schulten K (1996) VMD: visual molecular dynamics. *J Mol Graphics* 14:33-38.

Jain E, Bairoch A, Duvaud S, Phan I, Redaschi N, Suzek BE, Martin MJ, McGarvey P, Gasteiger E, (2009) Infrastructure for the life sciences: design and implementation of the UniProt website. *BMC Bioinformatics* 10:136.

Krivov GG, Shapovalov MV, Dunbrack Jr RL (2009) Improved prediction of protein side-chain conformations with SCWRL4. *Proteins*. 77*:*778-795.

Okur A, Wickstrom L, Simmerling C. (2008) Evaluation of Salt Bridge Structure and Energetics in Peptides Using Explicit, Implicit, and Hybrid Solvation Models *J. Chem. Theory Comput.* 4:488-498

Pettersen EF et al (2004) UCSF Chimera--a visualization system for exploratory research and analysis. *J Comput Chem.* 25:1605-1612.

Sali A, Blundell TL (1993) Comparative protein modelling by satisfaction of spatial restraints. *J Mol Biol* 234:779-815.

Shen M-Y, Sali A (2006) Statistical potential for assessment and prediction of protein structures. *Protein Sci* 15*:*2507-2524.

Waterhouse AM, Procter JB, Martin DMA, Clamp M, Barton G (2009) Jalview Version 2—a multiple sequence alignment editor and analysis workbench. *Bioinformatics* 25:1189-1191.
